# The disruption of the CCDC6 – PP4 axis induces a BRCAness like phenotype and sensitivity to PARP inhibitors in high-grade serous ovarian carcinoma

**DOI:** 10.1186/s13046-022-02459-2

**Published:** 2022-08-13

**Authors:** Francesco Morra, Francesco Merolla, Giovanna Damia, Francesca Ricci, Silvia Varricchio, Gennaro Ilardi, Laura Arenare, Daniela Califano, Virginia Napolitano, Robert Fruscio, Rosa Marina Melillo, Luca Palazzo, Angela Celetti

**Affiliations:** 1 Institute for the Experimental Endocrinology and Oncology, Research National Council, CNR, Naples, Italy; 2 Department of Medicine and Surgery, University of Milano-Bicocca, 20900 Monza, Italy; 3 Department of Medicine and Health Sciences “V. Tiberio”, University of Molise, Campobasso, Italy; 4 Department of Advanced Biomedical Sciences, Pathology Section, University of Naples “Federico II”, Naples, Italy; 5 Laboratory of Experimental Oncology, Department of Oncology, Istituto Di Ricerche Farmacologiche Mario Negri IRCCS, Milan, Italy; 6 Istituto Nazionale Tumori IRCCS, Fondazione G. Pascale, Naples, Italy; 7 Fondazione Policlinico “A. Gemelli”, IRCCS, 00168 Rome, Italy; 8 Clinic of Obstetrics and Gynecology, Department of Medicine and Surgery, San Gerardo Hospital, University of Milan Bicocca, Monza, Italy; 9 Department of Molecular Medicine and Medical Biotechnology, University of Naples Federico II, Naples, Italy

**Keywords:** Biomarker, PARP inhibitors, Cisplatin, Drugs resistance, PDX, PP4, H2AX phosphorylation

## Abstract

**Background:**

Treatment with PARP inhibitors (PARPi) is primarily effective against high-grade serous ovarian cancers (HGSOC) with *BRCA1/2* mutations or other deficiencies in homologous recombination (HR) repair mechanisms. However, resistance to PARPi frequently develops, mostly as a result of *BRCA1/2* reversion mutations. The tumour suppressor CCDC6 is involved in HR repair by regulating the PP4c phosphatase activity on γH2AX. In this work, we reported that in ovarian cancer cells, a physical or functional loss of CCDC6 results synthetic lethal with the PARP-inhibitors drugs, by affecting the HR repair. We also unravelled a role for CCDC6 as predictive marker of PARPi sensitivity in ovarian cancer, and the impact of CCDC6 downregulation in overcoming PARPi resistance in these tumours.

**Methods:**

A panel of HGSOC cell lines (either *BRCA*-wild type or mutant) were treated with PARPi after CCDC6 was attenuated by silencing or by inhibiting USP7, a CCDC6-deubiquitinating enzyme, and the effects on cell survival were assessed. At the cellular and molecular levels, the processes underlying the CCDC6-dependent modification of drugs’ sensitivity were examined. Patient-derived xenografts (PDXs) were immunostained for CCDC6, and the expression of the protein was analysed statistically after digital or visual means.

**Results:**

HGSOC cells acquired PARPi sensitivity after CCDC6 depletion. Notably, CCDC6 downregulation restored the PARPi sensitivity in newly generated or spontaneously resistant cells containing either wild type- or mutant-*BRCA2*. When in an un-phosphorylated state, the CCDC6 residue threonine 427 is crucial for effective CCDC6-PP4 complex formation and PP4 sequestration, which maintains high γH2AX levels and effective HR. Remarkably, the PP4-dependent control of HR repair is influenced by the CCDC6 constitutively phosphorylated mutant T427D or by the CCDC6 loss, favouring PARPi sensitivity. As a result, the PP4 regulatory component PP4R3α showed to be essential for both the activity of the PP4 complex and the CCDC6 dependent PARPi sensitivity. It's interesting to note that immunohistochemistry revealed an intense CCDC6 protein staining in olaparib-resistant HGSOC cells and PDXs.

**Conclusions:**

Our findings suggest that the physical loss or the functional impairment of CCDC6 enhances the PP4c complex activity, which causes BRCAness and PARPi sensitivity in HGSOC cells. Moreover, CCDC6 downregulation might overcome PARPi resistance in HGSOCs, thus supporting the potential of targeting CCDC6 by USP7 inhibitors to tackle PARPi resistance.

**Supplementary Information:**

The online version contains supplementary material available at 10.1186/s13046-022-02459-2.

## Background

Members of the poly(ADP-ribose) polymerase (PARP) superfamily of enzymes, specifically PARP1 and PARP2, are quickly recruited to DNA damage foci and initiate DNA damage response (DDR) after exposure to numerous DNA damage insults. Poly(ADP ribosyl)ation, also known as PARylation, is a posttranslational modification that is massively catalysed during DDR by the PARP1 and PARP2 proteins, assisted by the Histone PARylation Factor 1 (HPF1) protein [1, 2]. In response to DNA damaging insults, the transfer of ADP-ribose units from the donor NAD^+^ onto target proteins is essential for efficient repair, for instance by organising both chromatin rearrangements and the recruitment of DDR factors, such as ALC1, XRCC1, and LIG3 [3–6].

The enzymatic inhibition of DNA repair PARPs by small molecules has pioneered a synthetically lethal target therapy strategy for treatment of tumours characterised by DDR defects, such as breast and ovarian cancer carrying *BRCA1* and *BRCA2* mutations [7, 8]. Several PARP inhibitors (PARPi) have been developed and currently employed in the clinic or in clinical trials; these include olaparib, niraparib, rucaparib, veliparib, and talazoparib [9].

The inhibition of PARP1/2 enzymatic activity, coupled with PARP trapping, determines cells dependency on parallel pathways to maintain genome integrity, in particular homologous recombination (HR) [6, 7, 10, 11]. When HR is compromised, for example, due to inactivating mutations in *BRCA1* or *BRCA2*, cells are rendered superbly sensitive to PARPi [12–15]. The synthetic lethality strategy has been further extended to additional tumours with DDR defects, such as in *ATM*, *ATR*, and *PALB2* genes, and has shown impressive efficacy of PARPi in women with high-grade serous ovarian cancer (HGSOC), as maintenance treatment following platinum chemotherapy [7, 16, 17].

The most prevalent and deadly form of ovarian cancer, HGSOC, is frequently identified with extensive peritoneal metastases. Importantly, approximately 50% of HGSOCs are deficient in DDR, where germline or somatic mutations of *BRCA1* and *BRCA2* account for 20% of the cases; and *BRCA1/2* germline and somatic mutations represent clinically validated predictive biomarker of PARPi sensitivity [18]. In addition to *BRCA1* and *BRCA2* mutations, many other genetic alterations affecting DDR occur; *i.e.* the epigenetic silencing of *BRCA1* (11%), the amplification or mutation of *EMSY* (8%), the deletion of *PTEN* (7%), the hypermethylation of *RAD51C* (3%), mutations in *ATM* or *ATR* (2%) or mutations in FANC genes (5%) [19]. Overall, HGSOC has a mutational landscape that makes it susceptible to PARPi treatment.

Nevertheless, 20% of *BRCA1/2*-mutated ovarian malignancies do not respond to PARPi [20–23], highlighting the urgent need for new treatment approaches for inherently or acquired resistant tumours. The inhibition of poly(ADP-ribose) glycohydrolase (PARG), one of the enzymes necessary for reversing PARylation processes, shows promise as a treatment strategy for PARPi-resistant HGSOC [24–26]. It should be emphasised that HGSOC's susceptibility to PARPi and PARG inhibitors (PARGi) are mutually exclusive, which raises the possibility of a novel treatment approach for ovarian malignancies that are PARPi-resistant [24]. However, there is still a critical medical need for discovering novel biomarkers that can predict the sensitivity or resistance of HGSOC and/or alter the response of cancer to such medications when biomarkers are lost or acquired in tumours. One potential predictor and susceptibility factor for PARPi is CCDC6.

CCDC6 is a tumour suppressor protein known to be functionally lost due to gene translocations, somatic mutations, and altered protein levels, in several tumours [27]. CCDC6 translocates to the nucleus after DNA damage, where it participates to the HR via controlling H2AX phosphorylation following binding with the protein phosphatase PP4c [28, 29]. Notably, the balance between the E3 ubiquitin ligase FBXW7 and the de-ubiquitinase USP7 activities tightly controls CCDC6 protein levels and functions [30].

Here we unravelled a role for CCDC6 gene product in PARPi sensitivity of ovarian cancer cells via the PP4c-mediated modulation of HR proficiency. The physical or functional loss of CCDC6 could also overcome the acquired resistance to PARPi treatment in ovarian cancer patients. Altogether, our data suggest the potential of directly or indirectly targeting CCDC6 to tackle PARPi-resistance in HGSOCs.

## Methods

### Cell lines, drugs and chemicals

Ovarian cancer cell lines, OVCAR3, OV-90, PEO1 and PEO4 were purchased from ATCC (American Type Culture Collection) and were maintained, respectively, in RPMI (OVCAR3, PEO1 and PEO4) and DMEM (OV-90) (Gibco, Paisley, UK), supplemented with 20% (OVCAR-3) or 10% (OV-90, PEO1 and PEO4) fetal bovine serum (FBS; Gibco BRL, Italia), 1% L- Glutamine and 1% of penicillin – streptomycin (Gibco, Paisley, UK). Kuramochi cells were purchased from Sekisui XenoTech, LLC and were maintained in RPMI (Gibco, Paisley, UK), supplemented with 10% FBS, 1% L-Glutamine and 1% of penicillin – streptomycin (Gibco, Paisley, UK). Olaparib (AZD2281), and P005091 were provided by SelleckChem. PARG inhibitor (PDD00017273), SB-216763, cycloheximide, MG132 and cisplatin were provided by Merk Millipore. The genetic background of the ovarian cancer cells is reported in Additional File 1: Table S1.

### Generation of olaparib resistant (olaR) ovarian cancer cell lines

To generate OV-90 and OVCAR3 cells chronically resistant to olaparib (OV-90 olaR and OVCAR3 olaR), the cells were treated with an initial concentration of olaparib [2.5 µM] for 72 h. The treatment was followed by three times cells washing with Dulbecco’s phosphate-buffered saline (DPBS), followed by trypsinization and splitting. The surviving cells were grown to 80% confluence and split once a week over a period of at least two weeks to ensure viability. The concentration of drug was then sequentially increased to [5.0 μM] and [10 μM]. The OVCAR3 olaR and the OV-90 olaR cells viability was assessed by CellTiter 96 AQueous One Solution Cell Proliferation Assay, Promega (Madison, WI, USA).

### Reagents and antibodies

For the biochemical and immunofluorescence analysis the following antibodies were utilised: anti-CCDC6 (ab56353) Abcam, plc (Cambridge, UK), anti-tubulin (T6557), anti-PARG clone D8B10 (MABS61), anti-pan-ADP-ribose (MABE1016) Sigma-Aldrich, Inc, anti-PCNA (NANO3), anti-γH2AX (#05,636) Millipore (Burlington, MA, USA), anti-USP7 (A300-033A) Bethyl, Inc (Montgomery, TX, USA), anti-PARP (#9542) Cell Signaling, Inc (Danvers, MA, USA) and anti-Myc clone 9E10 (sc-40) Santa Cruz Biotechonology, Inc (Dallas, TX, USA). For the immunohistochemical analysis the anti-CCDC6 antibody (HPA 019,051) was from Merk Millipore. The secondary antibodies were from Biorad (Hercules, CA, USA).

### Sensitivity test and design for drug combination

Antiproliferative activity was determined by the CellTiter 96 AQueous One Solution Cell Proliferation Assay, Promega (Madison, WI, USA), in terms of 50% inhibitory concentration (IC50) values.

The cells were plated in triplicate in 96-well plates at a density of 1000 cells per well, and continuously exposed to each drug for 144 h. Each assay was performed in triplicate and IC50 values were expressed as mean ± standard deviation.

The results of the combined treatment were analysed by using the CompuSyn software [31]. The resulting combination index (CI) is a quantitative measure of the degree of interaction between different drugs. CI = 1 denotes additivity, CI > 1 denotes antagonism and CI < 1 denotes synergism.

### Protein extract and western blot analysis

Total cell extracts (TCE) were prepared with lysis buffer (50 mM Tris–HCl pH 7.5, 150 mM NaCl, 1% Triton X-100, 0.5% Na Deoxycholate, 0.1% SDS) and a mix of protease inhibitors. Protein concentration was estimated by a modified Bradford assay (Biorad). For Western blotting, cell lysates were separated by SDS-PAGE (10% polyacrylamide) and the proteins were transferred to a PVDF membrane. Membranes were blocked with 5% TBS-BSA and incubated with the primary antibodies. Immunoblotting experiments were carried out according to standard procedures and visualised using the ECL chemiluminescence system (Amersham, Little Chalfont, UK/Pharmacia Biotech, Milano, Italy). As a control for equal loading of protein lysates, the blotted proteins were probed with antibody against γ-tubulin.

### Plasmids and transfection

pcDNA4ToA-myc-CCDC6 plasmids were transfected with FuGene HD (Promega). From the PcDNA4ToA-myc-CCDC6 wild type template two myc-CCDC6 mutants (T427D and T427A), were created using the Quick-Change Site Directed Mutagenesis Kit, Agilent (Santa Clara, CA, USA), with the following primers: T427D Fw 5’-ttgggagatggaggcggatcgggccgtttgaattt-3’, T427D Rv 5’-aaattcaaacggcccgatccgcctccatctcccaa-3’; T427A Fw 5'-gatggaggcggcgcgggccgtttgaat-3', T427A Rv 5'-attcaaacggcccgcgccgcctccatc-3'. CCDC6 shRNA (pLKO.1 puro) was from Sigma-Aldrich, Inc. OV-90 and Kuramochi were transfected with a pool of non-targeting vector (ShCTRL) or with a plasmid pool (shCCDC6, NM_005436) for transient CCDC6 silencing by FuGene HD for 48 h.

For production of stable silenced cells, OVCAR-3 were transfected with ShCTRL or with ShCCDC6 plasmid pool by Nucleofection and selected with 0.5 μg/ml of Puromycin.

The siRNAs employed in this study were purchased from Sigma Aldrich. The RNAi transfections were performed using Oligofectamine Reagent (invitrogen). The PP4R3α siRNA oligo are: 5’-UGAAUUAAGUCGCCUUGAAUU-3’ and 5’-UUCAAGGCGACUUAAUUCAUU-3’.

The pDR-GFP reporter plasmid is based on a construct developed by M. Jasin [32] and contains two mutated GFP genes separated by a puromycin drug selection marker.

### Homologous recombination transient assay

Ovarian cancer cells were plated in a 60 mm plate and transfected with the pDR-GFP reporter alone (as negative control) or together with the pCAGGS-I-SceI plasmid. After 48 h cells were collected and analysed by FACS analysis with BD Accuri C6 Flow Cytometer (BD Bioscience, Canada).

The schematic representation of the pDR-GFP assay is shown in Additional File 2: Figure S1.

### Colony forming assay

10,000 cells (shCTRL or shCCDC6) per well were seeded into 6 well plates and continuously treated with the different drugs. After 10 days of incubation, prior to counting colonies, cells were fixed in methanol and stained with 0.5% Crystal Violet (10 min at room temperature). A population of at least 30 cells was scored as one survivable colony and considered for the count.

The colonies’ counting was performed at the optic microscope and through the open-source software ImageJ-NIH. The relative colony formation (percentage of colonies) was expressed as the number of colonies per treatment versus colonies that appeared in the DMSO control (mean colony counts ± standard errors are reported).

### TMA and IHC

A case series of 251 primary ovarian cancers was analysed on TMA. 6 TMAs were prepared with all the analysed samples [33]. Each tissue was examined for representability, and subsequently immunostained with anti-CCDC6 antibody (HPA 019,051, from Sigma-Aldrich), CCDC6 immunoreactivity of tumour cells was annotated. Visual annotation included staining intensity (negative, weak, moderate or strong), fraction of stained cells (% of total counted tumour cells), and subcellular localization. Following acquisition of anti-CCDC6 immunostained glass slides with a digital scanner, we also calculated the H-score on digital images by QuPath image analysis software [34, 35]. Immunohistochemistry was performed as already described [30]. Median H-score was 64.2 (IQR 28.8; 97.2); Mean H-score was 67.5 (sd 47.1). The frequency distribution of digitally evaluated CCDC6 H-Score is summarised in Additional File 3: Table S2. A summary of study population is shown in Additional File 4: Table S3.

PDX FFPE sample slides were immunostained with anti-CCDC6 antibody (HPA 019051, from Sigma-Aldrich) and IHC signal was quantified on digital slides with Positive Cell Count function of QuPath software. CCDC6 protein expression was reported as optical density arbitrary units. All the glass slides were digited with Aperio AT2 digital scanner (Leica).

### Cell blocks

Anti-CCDC6 immunostaining was performed on cell blocks-included OV-90 and OVCAR3 cells, parental (WT) and olaparib-resistant (olaR); PEO1 empty vector and myc-tagged CCDC6 overexpressing cells; PEO4 silenced for CCDC6 (shCCDC6) or control (shCTRL), as in plasmid and transfection paragraph.

Confluent cells, seeded in 100 mm plates, were collected and resuspended in 10% formalin and processed following 12 h fixation. According to manufacturer's instructions (7401150—Thermo Fisher Scientific, UK), collected samples were centrifuged for 1 min at 2.5 rpm and cell pellet resuspended in Reagent 2. Three drops of Reagent 1 were added into centre of Cytoblock cassette well and spun at 1500 rpm for 5 min. The Cytoblock cassette was processed in a standard tissue processor [36].

## Results

### CCDC6 protein levels contribute to the differential sensitivity to PARG and PARP inhibitors in high-grade serous ovarian carcinoma cell lines

The identification of tumour biomarkers that can predict sensitivity and/or resistance to PARPi as well as further enhance the selective cytotoxicity of such therapeutic drugs is highly envisaged in order to establish an accurate personalised medicine of HGSOC. According to preclinical research, attenuation of CCDC6 in several cancer cell cultures enhances sensitivity to PARPi, which have the ability to work in concert with cisplatin [36–41].

Here, we investigated if CCDC6 expression would influence how sensitive a panel of ovarian cancer cell lines was to PARPi and PARGi.

**Fig. 1:**
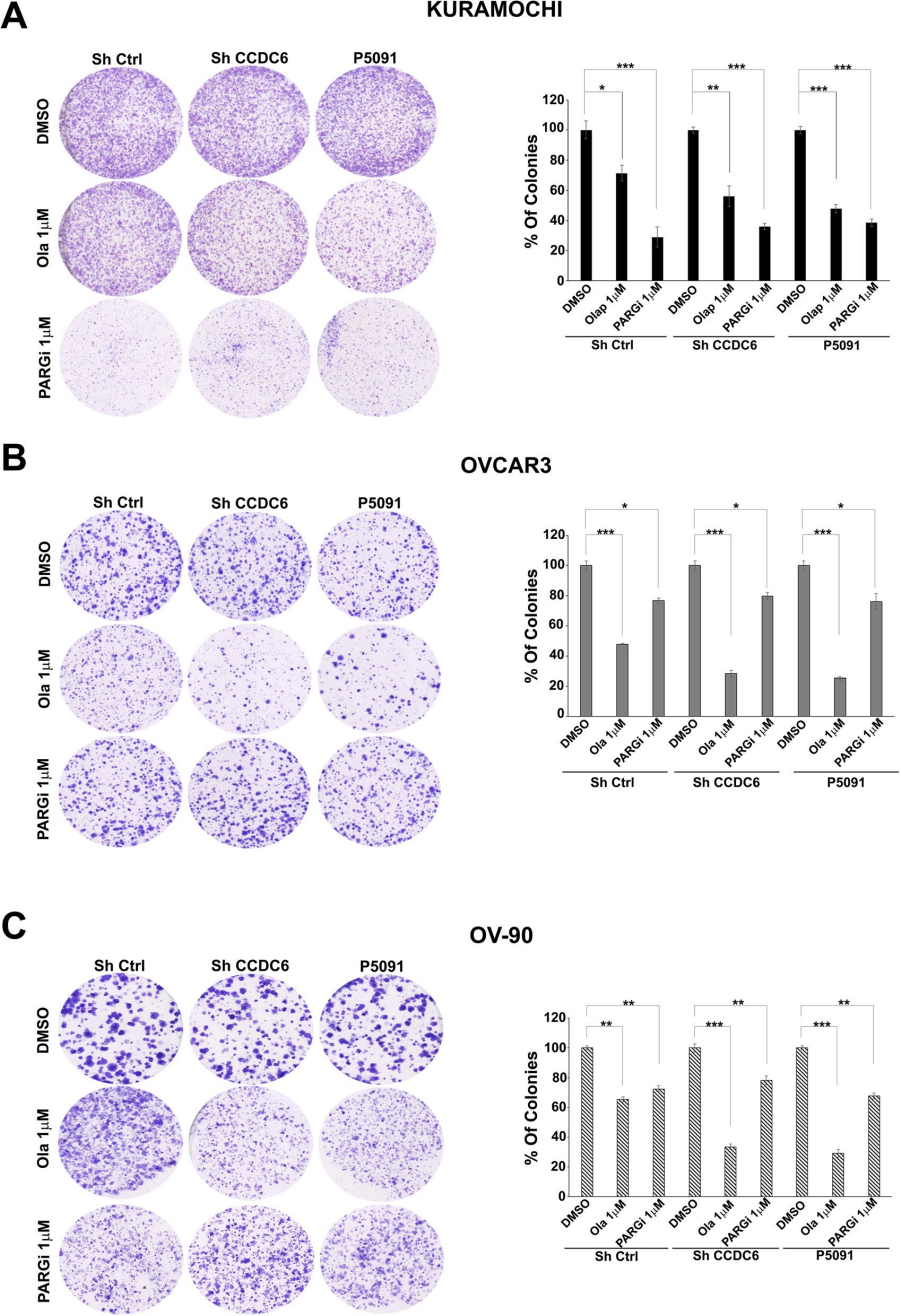
CCDC6 protein levels contribute to the differential sensitivity to PARPi and PARGi in HGSOC. **A-C** PARP-inhibitor olaparib [at 1 μM (Ola 1 μM)] and PARG-inhibitor [at 1 μM (PARGi 1 μM)] sensitivity in HGSOC cells: clonogenic assays were carried out in Kuramochi (**A**), OVCAR3 (**B**) and OV-90 (**C**) ovarian cancer cells silenced, upon transfection with short hairpin RNA for CCDC6 (ShCCDC6) or following treatment with P5091 [2.5 μM]. Scrambled shRNA (ShCtrl) or vehicle (DMSO) are used as controls. Cells were continuously exposed to DMSO, to 1 μM olaparib, or to 1 μM PARG-inhibitor PDD00017273. Colonies containing at least 30 cells were considered. The percentage of counted colonies were reported on the histograms on the right, for each cell line. Error bars indicate the measurement of the standard error mean, derived from three independent experiments. Statistical significance was verified by 2-tailed Student's t-test (* *p* < 0.05; ** *p* < 0.01 and *** *p* < 0.001)

To do so, in three ovarian cancer cell lines, namely Kuramochi, OVCAR3 and OV-90 cells, harbouring different genetic backgrounds (Additional File 1: Table S1) [42], we assessed relative sensitivity to the PARPi olaparib in colony formation assays: for these set of experiments we used a single representative drug concentration, as indicated [shCTRL] (Fig. 1A**-**C). The Kuramochi cells appeared minimally sensitive to PARPi (Fig. 1A) in comparison to the OVCAR3 and OV-90 cells (Fig. 1B, C), as supported by statistical analysis (Fig. 1A**-**C). The silencing of CCDC6 (shCCDC6) or pharmacological inhibition of the de-ubiquitinase USP7 (P5091), which results in proteasome-dependent degradation of CCDC6 [30] (Additional File 5: Figure S2A**-**F), determined a positive modulation of the PARPi sensitivity in all three cell lines (Fig. 1A**-**C).

Conversely, the Kuramochi cells resulted very responsive to the PARGi (PDD00017273) treatment, while OVCAR3 and OV-90 cells scarcely responded to PARGi (Fig. 1A**-**C) [24]. The low level of PARG and the high levels of ADP-ribosylated proteins, which are revealed by a pan-ADP-ribose antibody, supported the lower PARPi sensitivity and the higher PARGi response of Kuramochi cells, in comparison to OVCAR3 and OV-90 cells [6] (Additional File 5: Figure S2 G, H). In a 2**-**D colony-forming assay with one concentration drug, CCDC6 silencing diminished sensitivity to PDD00017273 in Kuramochi cells (Fig. 1A). By contrast, the CCDC6 attenuation minimally affected the PARGi sensitivity in OVCAR3 and OV-90 cells (Fig. 1B**-**C). Nevertheless, when these ovarian cancer cells were challenged with different concentration of PARGi and their vitality assessed by MTT assays, the change of PARGi sensitivity upon CCDC6 downregulation appeared evident, as described below.

The relative CCDC6 and USP7 levels in all the analysed ovarian cancer cells are shown in Additional File 5: Figure S2G.

Together these results demonstrate that the genetic or pharmacological modulation of the CCDC6 protein levels in ovarian cancer cells affects sensitivity to the anti-cancer PARPi as well as to the recently developed PARGi.

### CCDC6 depletion affects γH2AX foci formation in response to PARP and PARG inhibitors and impairs double strand breaks repair by Homologous Recombination

**Fig. 2:**
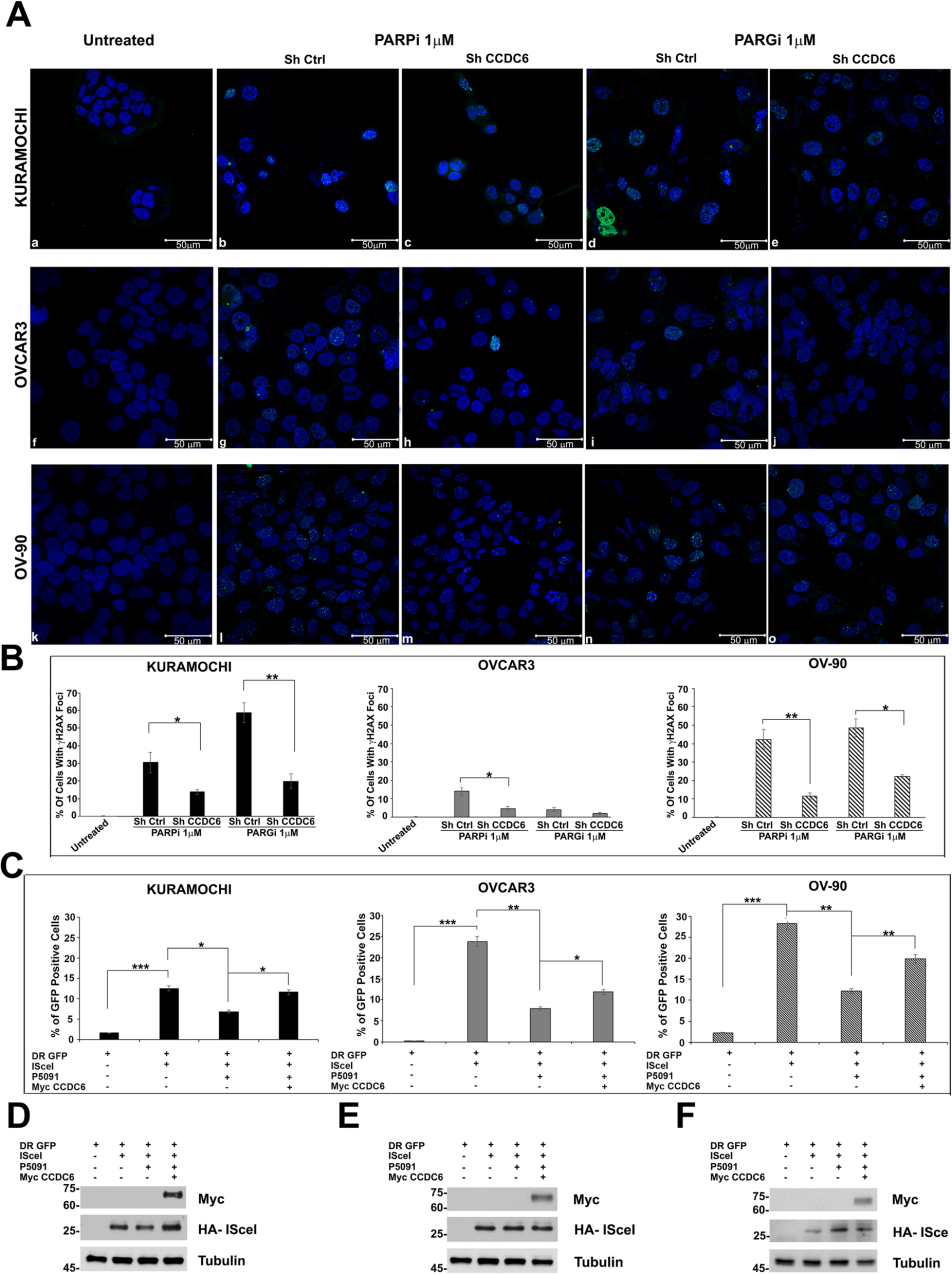
CCDC6 depletion affects γH2AX foci formation and impairs Homologous Recombination repair. **A** Immunofluorescence images showing, compared to untreated control cells (a,f,k), the impact of CCDC6 silencing, followed by exposure to PARPi [1 μM] (b,c,g,h,l,m) or PARGi [1 μM] (d,e,i,j,n,o) for 48 h, on γH2AX nuclear foci formation in Kuramochi, OVCAR3 and OV-90 cells. Scale bar 50 μm. **B** Graphs represent the percentage of cells with more than 15 foci. Error bars indicate the standard error mean derived from three independent experiments. Statistical significance was verified by 2-tailed Student's t-test (* *p* < 0.05; ** *p* < 0.01 and *** *p* < 0.001). **C** Kuramochi, OVCAR 3 and OV-90 cells, after pre-treatment either with vehicle or P5091 [2.5 μM] for 4 h, were transfected with DR-GFP alone, as control, with HA-ISceI (ISceI) and with both HA-ISceI (ISceI) and Myc CCDC6 plasmids. The percentages of GFP positive cells, compared to controls, were plotted as histograms, representative of the mean of three independent experiments. Error bars indicate the measurement of the standard error mean. Statistical significance was verified by 2-tailed Student's t-test (* *p* < 0.05; ** *p* < 0.01 and *** *p* < 0.001). **D**, **E**, **F** Myc CCDC6 and HA-ISceI proteins expression were assessed by anti-Myc and anti-HA antibodies at Western Blot. Anti-Tubulin immunoblots are shown as loading control

We evaluated the formation of γH2AX foci, a marker for double strand breaks (DSBs), in order to thoroughly study the molecular mechanisms of CCDC6-dependent effects on the susceptibility of HGSOC cells to PARPi and/or PARGi [43, 44]. In contrast to the untreated cells, foci formation was significantly induced by both PARPi and PARGi in the Kuramochi and OV-90 cell lines, and CCDC6 silencing significantly reduced these effects. In comparison, PARPi and PARGi had minimal effect on γH2AX foci in control OVCAR3 (shCTRL) cells. However, CCDC6 silencing significantly reduced PARPi-induced foci formation in these cells (Fig. 2A, B). Kuramochi cells responded dramatically to the addition of the PARGi therapy, as previously described [24].

Strikingly, silencing of CCDC6 resulted in a considerable reduction in γH2AX foci in all three HGSOC cell lines as seen in the Figure and by relative intensity quantification (Fig. 2A, B). Notably, over-expression of myc-CCDC6 plasmid rescued γH2AX foci formation in CCDC6-downregulated cells, thus excluding off-target issues (Additional File 6: Figure S3, Additional File 7: Figure S4, Additional File 8: Figure S5).

The drugs’ effect observed upon CCDC6 silencing suggested an impairment of DSBs repair by HR in the analysed ovarian cancer cells. In order to assess the efficacy of the HR repair, we employed the DR-GFP reporter system [32]. A schematic representation of the DR-GFP assay is shown in Additional File 2: Figure S1. In these assays, we decided to restrain endogenous CCDC6 activity by using the pharmacologic inhibitor P5091. The ovarian cancer cells, left untreated or after pre-treatment with P5091, were transfected with the DR-GFP reporter plasmid alone, as a control, or together with the I-SceI plasmid able to induce DSBs. The ability to repair the DSBs by HR was measured by flow cytometry and the frequency of HR was reported as a percentage of GFP positive cells. Treatment with USP7 inhibitor determined a significant decrease of the GFP positive cells, compared to non-treated cells, suggesting that the reduction of CCDC6 levels affected the DNA repair by HR in all the ovarian cancer cells analysed, even if the effects were most evident in OVCAR3 and OV-90 cells, with respect to Kuramochi cells (Fig. 2C). The phenotype induced by the P5091 treatment was mainly dependent on the CCDC6 increased turnover, as it was almost completely (Kuramochi) or partially (OVCAR3 and OV-90) reverted following the myc-CCDC6 transient transfection (Fig. 2C). In ovarian cancer cells the transfection efficiency of HA-ISceI, in presence or absence of the myc-CCDC6 plasmid, was assessed by western blot (Fig. 2D**-**F).

These data suggest that CCDC6 loss of function confers an HR-deficiency phenotype in HGSOC cancer cells.

### CCDC6 loss sensitises the high-grade serous ovarian carcinoma cell lines to combined treatment with PARP inhibitors and cisplatinum

**Fig. 3:**
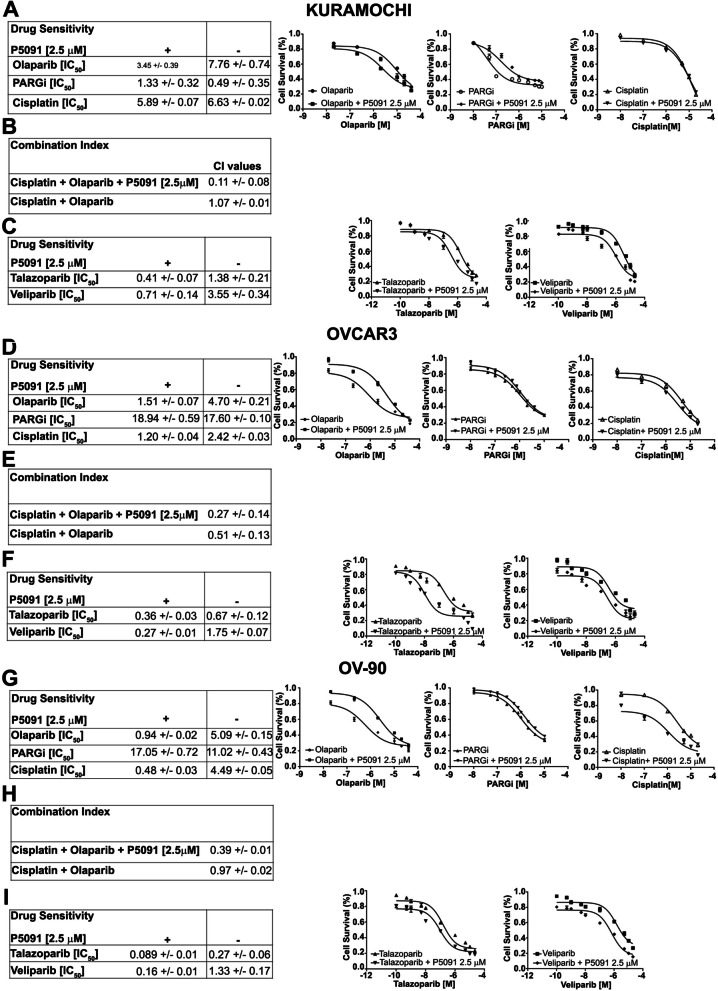
The chemical depletion of CCDC6 gene product by P5091, sensitizes ovarian carcinoma cells to the PARP inhibitor olaparib alone or in combination with cisplatin. (**A**, **D**, **G**, left side): Drugs sensitivity to Olaparib, PARG inhibitor (PARGi) and Cisplatin, in presence or absence of P5091 [2.5 μM] was determined by a modified 3-(4,5-dimethylthiazole-2- yl)-2–5-diphenyltetrazolium bromide assay, CellTiter 96 Aqueous One Solution assay (Promega), and was expressed as 50% inhibitory concentration (IC50) values. (**A**, **D**, **G**, right side): Surviving fractions of Kuramochi, OVCAR3 and OV-90 cells treated for 144 h, in presence or absence of P5091 [2.5 μM], with different doses of Olaparib, PARG inhibitor and Cisplatin. (**B**, **E**, **H**) The combination index values (CI) according to 1:2 concentration ratio of Cisplatin and Olaparib, in presence or absence of P5091 [2.5 μM] are shown. (**C**, **F**, **I**, left side): Drugs sensitivity to the PARP inhibitors Talazoparib and Veliparib, in presence or absence of P5091 [2.5 μM] are displayed. (**C**, **F**, **I**, right side) Surviving fractions of Kuramochi, OVCAR3 and OV-90 cells treated for 144 h, in presence or absence of P5091 [2.5 μM], with different doses of Talazoparib and Veliparib, were determined and expressed as in (**A**, **D**, **G**, right side)

Since the HR deficiency is also accompanied by sensitivity to PARPi, we used cellular metabolic activity as a marker of cell survival to characterize and quantify the effects of CCDC6 downregulation on olaparib sensitivity in HGSOC. To do this, we reduced CCDC6 protein levels in ovarian cancer cells using the USP7 inhibitor P5091 and evaluated sensitivity to various olaparib concentrations. Similarly, PARGi and cisplatin sensitivity were also assessed (Fig. 3A**-**I). Generally, CCDC6 downregulation by P5091 improved sensitivity to olaparib in all cell lines herein analysed. Specifically, the sensitivity to olaparib in Kuramochi cells (IC50 7.76 μM ± 0.74) was positively modulated to 3.45 μM ± 0.39 by the CCDC6 chemical attenuation (Fig. 3A). Stronger cytotoxic effects were attained in P5091-pretreated OVCAR3 and OV-90 cells when exposed to olaparib compared to Kuramochi cells; these cells' sensitivity increased by about three and five times, respectively (Fig. 3D, G). Similarly, genetic downregulation of CCDC6 by shRNA leads to overlapping improved sensitivity to olaparib in all cellular models herein analysed (Additional File 9: Figure S6A, D, G).

Platinum containing drugs are widely used for the treatment of ovarian cancer. However, ovarian cancer patients initially responsive to platinum containing drugs invariably relapse becoming resistant. Here we asked whether CCDC6 downregulation may affect the vulnerability of HGSOC to cisplatin alone or in combination with olaparib. While sensitivity to cisplatin was only slightly affected by P5091 (IC50 5.89 μM ± 0.07) in Kuramochi cell lines compared to vehicle (IC50: 6.63 μM ± 0.02) (Fig. 3A), CCDC6 chemical downregulation significantly modifies cisplatin sensitivity in OVCAR3 (IC50 2.42 μM ± 0.03 vs IC50 1.20 μM ± 0.04) and OV-90 cells (IC50 4.49 μM ± 0.05 vs IC50 0.48 μM ± 0.03) (Fig. 3D, G ). Strikingly, in these cells the combined treatment of olaparib with cisplatin showed a synergistic effect, magnified by the CCDC6 accelerated turnover, upon P5091 addition (Fig. 3E, H).

Conversely, sensitivity to PARGi in CCDC6 downregulated cells behaved differently compared to olaparib treatment, which is explained by their different mechanism of action (as discussed later in the text). As reported, Kuramochi cells resulted highly responsive to the PARGi treatment (IC50 0.49 μM ± 0.35). Notably, the CCDC6 downregulation by P5091 treatment affected PARGi-induced cytotoxicity (in the presence of P5091 at 2.5 μM, the IC50 raised to 1.33 μM ± 0.32) (Fig. 3A). Similar results were obtained by silencing CCDC6 by shRNA (Additional File 9: Figure S6A). The CCDC6-dependent weak response to PARGi was also visible in intrinsically PARGi-resistant OVCAR3 and OV-90 cell lines, where the low sensitivity to PARGi was enhanced by P5091 (Fig. 3D, G). In detail, following the PARGi addition, the CCDC6-depleted OV-90 cells showed an IC50 of 17.05 μM ± 0.72 compared with an IC50 of 11.02 μM ± 0.43 of control cells. The CCDC6-depleted OVCAR3 cells also exhibited a mild modulation showing an IC50 of 18.94 μM ± 0.59 compared with an IC50 of 17.60 μM ± 0.10 of control cells (Fig. 3D, G ). Importantly, all these phenotypes were rescued by CCDC6 exogenous expression in CCDC6 chemically or genetically downregulated cell lines (Additional File 9: Figure S6 A**-**B, D**-**E and G**-**H), thus supporting the specificity of our observations. In detail, in Kuramochi-CCDC6-depleted cells the re-expression of CCDC6 (Additional File 9: Figure S6B) led to an IC50 of 0.76 μM ± 0.10 vs an IC50 of 1.11 μM ± 0.08 detected in control cells (transfected with the empty vector, EV); in OVCAR3 CCDC6 silenced cells the re-expression of CCDC6 (Additional File 9: Figure S6E) determined an IC50 of 18.52 μM ± 0.08 vs an IC50 of 19.36 μM ± 0.05 relieved in control cells; and in OV-90 cells CCDC6-depleted, the re-expression of CCDC6 (Additional File 9: Figure S6H) led to an IC50 of 15.08 μM ± 0.07 vs an IC50 of 17.80 μM ± 0.02 detected in control cells (transfected with the empty vector, EV). By contrast, the overexpression of CCDC6 in wild type Kuramochi, OV-90 and OVCAR3 cells did not determine any variation in the IC50 values upon treatment with different doses of PARGi (Additional File 9: Figure S6B, E, H).

By subjecting HGSOC cells to various PARPi with varied trapping and inhibitory potencies, we further tested whether the increased sensitivity to olaparib could be replicated [45]. Both talazoparib and veliparib, PARP inhibitors with greater PARP activity inhibition and trapping action, have recently received clinical approval for breast cancer treatment, enabling dosage reduction and improving clinical efficacy [17]. Therefore, we examined their effectiveness alone or in conjunction with P5091 in our HGSOC preclinical models. Intriguingly, pharmacological downregulation of CCDC6 enhances the cytotoxic effects of both PARPi in all three cellular models, with P5091-treated OVCAR3 cells showing the most impressive 18.6-fold increase in talazoparib sensitivity (Fig. 3C, F, I).

These findings imply that CCDC6 downregulation enhanced HGSOC cell line sensitivity to various PARPi, regardless of their trapping and inhibitory efficacy.

### CCDC6 downregulation rescues the sensitivity to olaparib in newly generated PARP inhibitor resistant ovarian cancer cells

**Fig. 4:**
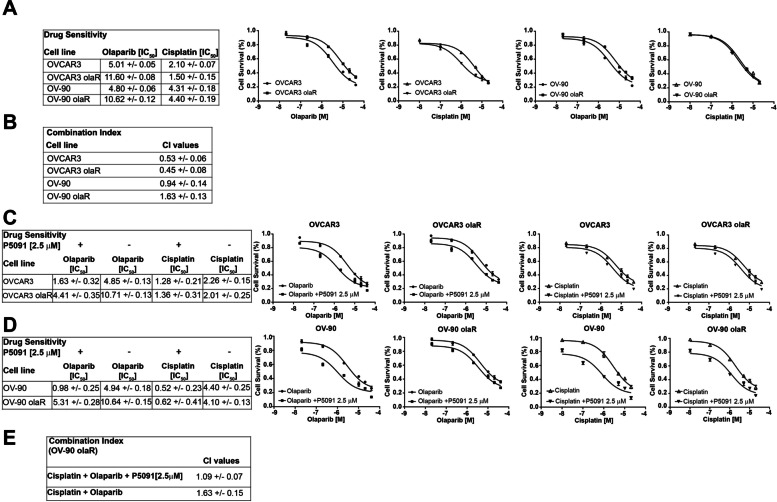
CCDC6 downregulation can restore the olaparib sensitivity in resistant ovarian cancer cells. **A** Drugs sensitivity and surviving fraction in OVCAR3 olaparib Resistant (OVCAR3 olaR) and OV-90 olaparib Resistant (OV-90 olaR) cells, compared to the parental OVCAR3 and OV-90 cells, after 144 h exposure to different doses of Olaparib and Cisplatin. **B** In the figure, the CI values, according to 1:2 concentration ratio of Cisplatin and Olaparib, are reported. **C**, **D** IC50 values and surviving fraction of OVCAR3 olaR and OV-90 olaR cells, compared to parental OVCAR3 and OV-90 cells, after 144 h exposure to Olaparib and Cisplatin, in presence or absence of P5091, are displayed. **E** In OV-90 olaR, the CI values according to 1:2 concentration ratio of Cisplatin and Olaparib, in presence or absence of P5091 [2.5 μM] are shown

Our results suggest the potential of predicting PARPi sensitivity in HGSOCs harbouring CCDC6 loss of functions as well as of downregulating CCDC6 by USP7 inhibitor, even in PARPi-resistant, *BRCA* wild type (WT), HR competent HGSOC cells. In order to address the intriguing properties of CCDC6 downregulation in re-sensitising PARPi-resistant HGSOC, we generated olaparib-resistant OVCAR3 and OV-90 cell lines, thereafter named OVCAR3olaR and OV-90olaR, respectively, by exposing cell lines to PARPi at IC50 doses and up to 10 μM for two weeks, as described in material and methods section. Compared to the parental OVCAR3 and OV-90 cells, the cytotoxic effects of the PARPi treatment were quantified in the newly generated OVCAR3olaR and the OV-90olaR by cell viability assays (Fig. 4A). Of note, compared to parental cells, CCDC6 protein levels were slightly increased in OV-90olaR at IHC and western blot, while resulted comparable in PARPi-resistant OVCAR3 cells (Additional File 10: Figure S7 D**-**G). However, the CCDC6 attenuation upon the P5091 treatment, rescued the sensitivity to PARPi in OVCAR3olaR and OV-90olaR HGSOC cells (Fig. 4C, D).

The generation of the olaparib resistance phenotype left nearly unaffected the cisplatin sensitivity in these cells (Fig. 4A). However, while in OV-90olaR cells the combined treatment of cisplatin and olaparib resulted in an antagonistic effect (CI > 1), in OV-90 parental cells it determined a synergistic effect (CI < 1) (Fig. 4B). Interestingly, in the presence of P5091, which increases the CCDC6 degradation, the combined treatment of cisplatin and olaparib turned into an additive effect in OV-90olaR cells (CI = 1) (Fig. 4E). No variations in the combination index between OVCAR3 parental and resistant cells upon cisplatin and olaparib treatment were registered (Fig. 4B).

**Fig. 5:**
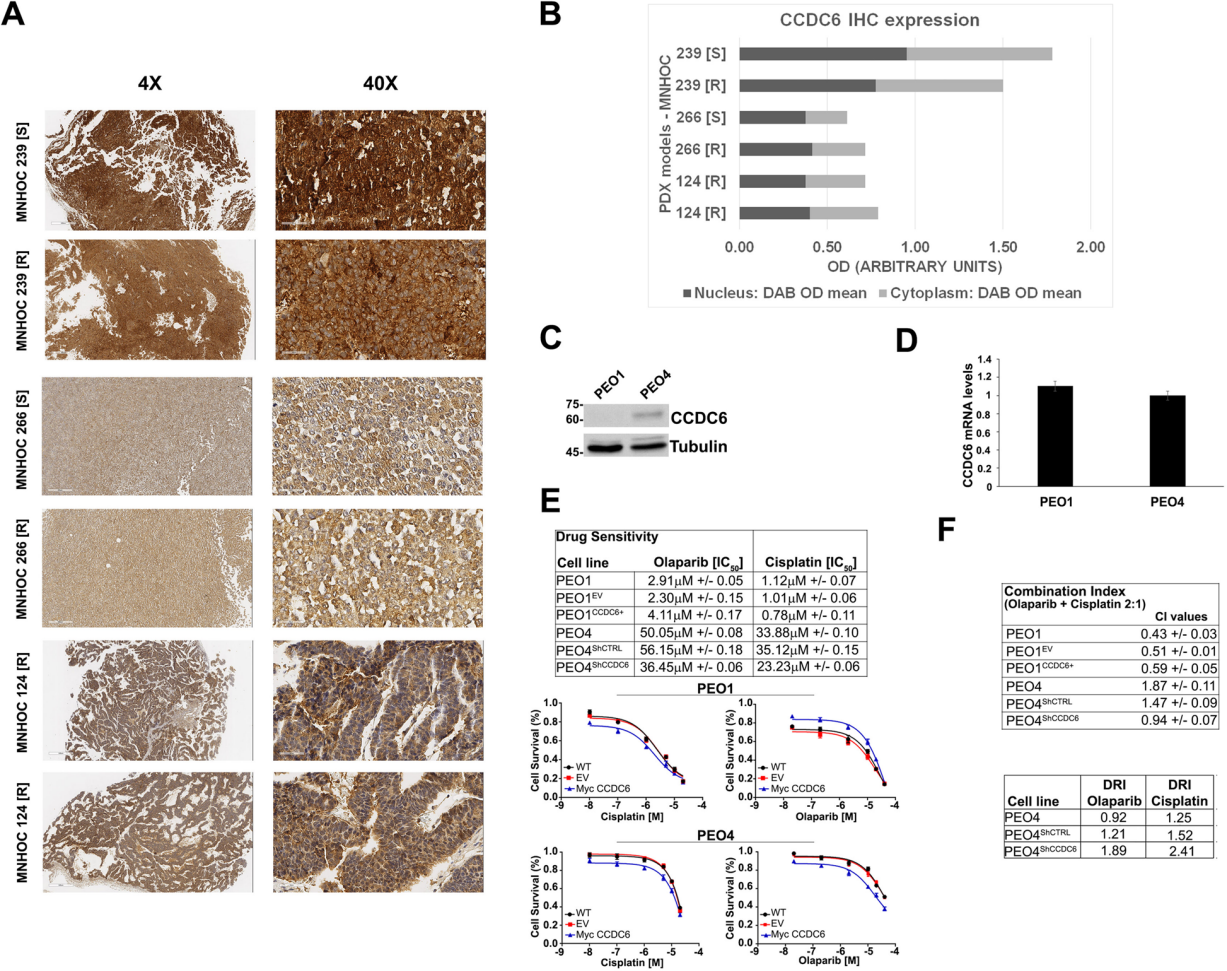
CCDC6 protein levels evaluation in Patient Derived Xenograft (PDX) and in naturally occurring CCDC6-null and CCDC6-competent HGSOC models**. A** MNHOC 239, MNHOC 266, and MNHOC 124 PDX samples were immunostained with an anti-CCDC6 antibody. IHC analysis revealed a diffuse positivity in all the analysed samples with negligible differences between them. ([S]: cisplatin sensitive; [R]: cisplatin resistant). **B** CCDC6 IHC expression on PDX samples was quantified on digital glass slides and reported as Optical Density (OD) arbitrary units. The graph shows Nuclear and Cytoplasmatic CCDC6 expression in each analysed sample. **C** CCDC6 protein expression levels in PEO1 and PEO4 cells are shown by anti-CCDC6 and anti-tubulin immunoblots. **D** CCDC6 relative mRNA expression in PEO1 and PEO4 cells as evaluated by quantitative RT-PCR. **E** Olaparib and Cisplatin sensitivity in CCDC6-naturally null PEO1 cells and in CCDC6-proficient PEO4 cells have been evaluated. The rescue with myc-CCDC6 in PEO1 cells (CCDC6 +) and the silencing by ShCCDC6 in PEO4 cells, modulated the drugs sensitivity with respect to controls (EV or ShCTRL). IC50 values and surviving fractions are displayed. **F** CI values according to 1:2 concentration ratio of Cisplatin and Olaparib and the Dose Reduction Index (DRI) for each drug are reported in the tables. In the PEO4 cells, following CCDC6 depletion, the DRI resulted > 1, as the dose of Olaparib and Cisplatin to obtain the 50% Fractional Effect (IC50) resulted reduced. Error bars indicate the standard error mean derived from three independent experiments

Then, by hypothesizing CCDC6 as a possible biomarker of cisplatin response, we investigated the CCDC6 expression levels in three ovarian patient-derived xenografts (PDXs) couples, sensitive and with acquired resistance to cisplatin (MNHOC 266, MNHOC 124 and MNHOC 239), obtained after multiple in vivo drug treatment [46–48]. In particular, the PDX MNHOC 266, carries mutations in *BRCA1*, whereas the PDXs MNHOC 124 and MNHOC 239 are *BRCA1/2* WT. The models recapitulate the clinical setting of cisplatin resistance. Following an immunohistochemical staining to assess the levels of CCDC6, a strong expression was found in all PDXs (Fig. 5A, B), with a pattern of CCDC6 staining that was comparable in sensitive and the matching cisplatin resistant PDXs.

Although the CCDC6 staining data on PDXs do not discriminate a primary from a resistant ovarian tumour model, we may hypothesise, based on in vitro evidence, that the CCDC6 positive staining could otherwise predict the response to PARPi, as described in other tumour models [27].

All the PDXs were non-responders to the olaparib treatment. They reproduced the phenotype of the newly generated olaparib-resistant ovarian cancer cells, where CCDC6 showed a high level of expression, mostly mimicking the PDX phenotype (Fig. 5, Additional File 10: Figure S7 D**-**E).

We used the well-known PEO1 and PEO4 cells [49], which represent naturally occurring CCDC6 null and CCDC6 competent ovarian cancer cell types, respectively, that display differing protein levels despite having equivalent amounts of CCDC6 transcripts, to test this hypothesis (Fig. 5C, D). These cells were from the same patient who had a poorly differentiated serous adenocarcinoma and who after chemotherapy had a status change from platinum sensitivity (PEO1) to platinum resistance (PEO4) [49].

Notably, the PEO1 cells are the first and only human BRCA2 defective ovarian cancer cell line identified thus far and, like the original patient, possess a BRCA2 hemizygous nonsense mutation 5193C > G (Y1655X). PEO4 cells, derived from ascites at the time of relapse with cisplatin resistance, have the secondary mutation (Y1655 mutation spontaneously reverted) and are *BRCA2* proficient.

Following CCDC6 knockdown by shRNA in the cisplatin-resistant PEO4 primary cells, the cytotoxic effects of olaparib and the cisplatin sensitivity were measured. The very low sensitivity to olaparib in the PEO4 cells (IC50, 56.15 μM ± 0.18) was positively modulated by the CCDC6 lowering (IC50 dropped to 36.45 μM ± 0.06) (Fig. 5E); strikingly, in these cells, the cisplatin response (IC50 35.12 μM ± 0.15) was also positively modulated by the CCDC6 attenuation (IC50 23.23 μM ± 0.06) (Fig. 5E, Additional File 10: Figure S7A). Most importantly, upon CCDC6 depletion, the combined treatment of cisplatin and olaparib determined a synergistic effect (CI < 1), while an antagonistic effect (CI > 1) was observed in the PEO4 parental cells (Fig. 5F). Remarkably, the dose response index (DRI) was impressively modulated by the drug combination in PEO4 cells, silenced for CCDC6 (DRI > 1), since the IC50 concentration dropped by more than 30% for both the drugs concentration (Fig. 5F).

Notably, the PEO1 cells resulted naturally null for the CCDC6 protein expression, although CCDC6 mRNA is well expressed in these cells and in PEO4 cells. This suggests that post-translational processes are responsible for CCDC6 deregulation in PEO1 cells. According to preliminary results (Morra F et al., in preparation), the low amount of CCDC6 protein detected in PEO1 cells might be ascribed to GSK3β gain of activity, which is common in *BRCA2*-mutant cells [50], in turn able to sustain the FBXW7-dependent CCDC6 degradation [30]. Importantly, CCDC6 protein levels spontaneously re-established in *BRCA2* reverted PEO4 cells (Fig. 5C). The CCDC6 null, cisplatin sensitive, PEO1 cells showed a high PARPi sensitivity (IC50: 2.3 μM ± 0.15). This phenotype was abolished by replenishing the expression of CCDC6, as observed by a 70% increase of the IC50 drug concentration (IC50: 4.11 μM ± 0.17) (Fig. 5E). Moreover, cisplatin sensitivity remained nearly unaffected in the presence of CCDC6 ectopic expression (in PEO1 CCDC6 + : IC50 0.78 μM ± 0.11 vs. IC50 1.01 μM ± 0.06 in PEO1 EV cells). However, upon cisplatin and olaparib combined treatment, no significant variations in the combination index between the PEO1 cisplatin sensitive cells, overexpressing either the Myc empty vector (EV) or the Myc CCDC6 plasmid (CCDC6 +), were registered. Thus, CCDC6 expression affects olaparib, but not cisplatin sensitivity of these cells. Furthermore, the ability to repair the DNA DSBs by HR was determined in the PEO1 cells by GFP reporter assays, which revealed a slightly increase in the GFP positive cells following the CCDC6 transient transfection [Myc CCDC6 plasmid (CCDC6 +)], in comparison to the control cells [Myc empty vector (EV)] where a very low percentage of GFP positive cells was detected, mostly ascribed to the presence of *BRCA2* inactivating mutation (Additional File 10: Figure S7A-C).

This observation indicates that, in the context of BRCA2 deficiency, CCDC6 activity in HR repair is limited and is epistatic to BRCA2. Nevertheless, in the PEO4 cells, in which BRCA2 functional activity and CCDC6 levels are restored, the CCDC6 depletion determined a significant decrease of GFP positive cells, compared to the control HR proficient cells (Additional File 10: Figure S7B-C).

Overall, our results imply that assessing CCDC6 levels in tumours may provide critical information for therapy choices in HGSOC.

### The CCDC6-PP4c interaction depends on phosphorylation and determines PARP inhibitors sensitivity by modulating γH2AX levels

To further investigate the molecular mechanisms of PARPi improved sensitivity in HGSOC where CCDC6 is downregulated, we dissected CCDC6 roles in HR response to DNA damage. Indeed, by regulating histone H2AX phosphorylation status, CCDC6 contributes to efficient DDR through HR. Upon DNA damage exposure and in an ATM-dependent manner, CCDC6 moves from cytosol to the nucleus where it binds the main phosphatase responsible for the maintenance of histone H2AX phosphorylation status, PP4c [28, 51]. The PP4 holoenzyme, consisting of the catalytic subunit (PP4c) and the two major isoforms of PP4R3 (PP4R3α/β, also known as SMEK1/2) binds to the F^423^xxP^426^ motif in CCDC6, providing specificity [29]. Moreover, the residue Threonine 427, close to the motif, can modulate the CCDC6 binding to PP4 and determine the CCDC6 intracellular localization through its phosphorylation status [29]. In order to exploit the functional outcome of the Threonine 427, flanking the FxxP interaction motif and preventing, when phosphorylated, the binding to PP4, we performed site-directed mutagenesis of this residue in alanine (T427A) or aspartate (T427D). We investigated whether the disruption of CCDC6-PP4c interaction might affect the histone H2AX phosphorylation leading to HR deficiency and to PARPi sensitivity.

**Fig. 6:**
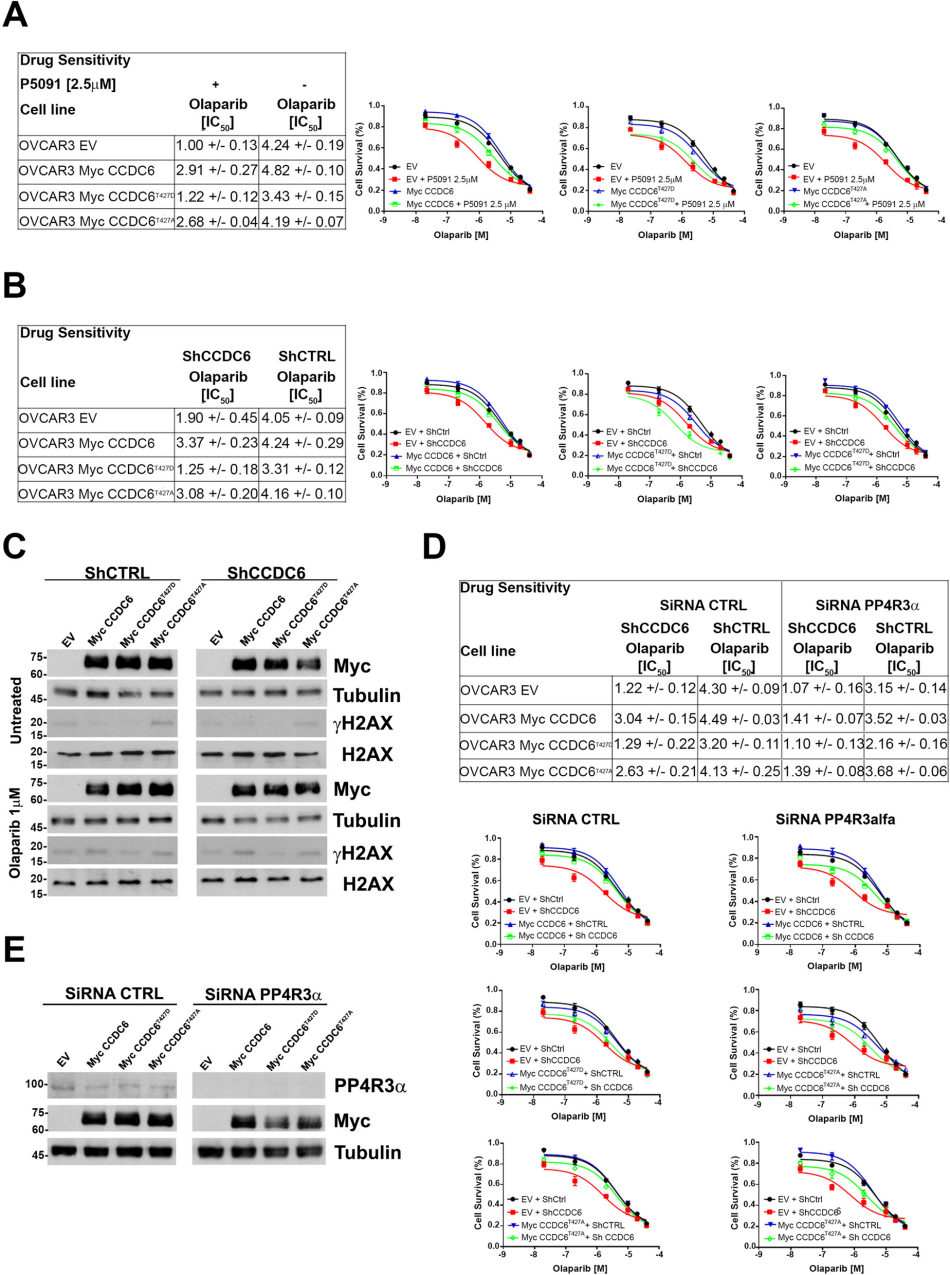
The CCDC6 threonine 427 and the regulatory subunit PP4R3α turned up critical for the CCDC6-dependent PARP inhibitors sensitivity. **A**, **B** In OVCAR3 cells depleted for CCDC6, upon treatment with P5091 [2.5 μM] or transfection with short hairpin RNA for CCDC6 (ShCCDC6), the Olaparib sensitivity has been evaluated following transient expression of CCDC6 wild type (Myc CCDC6), CCDC6 mutants (Myc CCDC6^T427A^, Myc CCDC6^T427D^) or empty vector (EV), at the indicated doses. Scrambled shRNA (ShCTRL) or vehicle were used as control. **C** OVCAR3 cells stably silenced for CCDC6 (ShCCDC6), and control cells (ShCTRL), were transfected with CCDC6 wild type, CCDC6 mutants or empty vector as in (**A**, **B**) and treated with olaparib [1 μM]. Equal amounts of cell lysates were immunoblotted with anti-γH2AX and H2AX antibodies. Anti-Myc and anti-Tubulin antibodies were employed as transfection and loading controls, respectively. **D** OVCAR3 cells, stably depleted for CCDC6 (ShCCDC6) or control (ShCTRL) were transfected with siRNA for PP4R3α and the olaparib sensitivity was assessed upon overexpression of CCDC6 wild type, CCDC6 mutants or empty vector, as in (**A**, **B**). **E** The efficacy of PP4R3α silencing or of Myc CCDC6 overexpression were assessed at Western Blot by the anti-PP4R3α or anti-Myc antibodies. Anti-Tubulin immunoblots are shown as loading control. In (**A**, **B** and **D**) the drugs sensitivity was determined by a modified 3-(4,5-dimethylthiazole-2- yl)-2–5-diphenyltetrazolium bromide assay, CellTiter 96 Aqueous One Solution assay (Promega), and was expressed as 50% inhibitory concentration (IC50) values. On the right side the surviving fractions of the OVCAR3 cells, as in (**A**, **B** and **D**), treated for 144 h with different doses of Olaparib, are shown

In HGSOC cells, pharmacologically or genetically silenced for CCDC6, and upon treatment with different concentrations of olaparib, as indicated, the cytotoxic drug effects were quantified by a cell viability assay upon overexpression of myc-CCDC6WT (wild type), CCDC6T427A, CCDC6 T427D or empty vector (EV), as control (Fig. 6A). The olaparib sensitivity observed in the OVCAR3 stably silenced for CCDC6, and transiently transfected with the EV, (IC50: 1.90 μM ± 0.45), decreased following the transient transfection of myc-CCDC6WT (IC50: 3.37 μM ± 0.23) and myc-CCDC6T427A (IC50: 3.08 μM ± 0.20) plasmids; this data suggests that the T427A mutant, as well as the wild type protein, maintains the ability to interact with PP4c and to inhibit its phosphatase activity toward histone H2AX [29]. However, even after forced expression of the CCDC6T427D mutant, OVCAR3 remains sensitive to olaparib whether treated with the USP7i P5091 or stably silenced for CCDC6, indicating that this mutant is unable to engage with PP4c phosphatase (Fig. 6A, B). As seen in the western blot analysis, this causes an increase in PP4c activity and a corresponding decrease in histone H2AX phosphorylation, which reproduces the CCDC6 deletion phenotype (Fig. 6C). γH2AX levels were assessed in olaparib-treated (1 μM) or untreated CCDC6-silenced ovarian cancer cells by western blot. This analysis revealed lower γH2AX levels in cells overexpressing the T427D mutant with respect to those expressing the T427A mutant or the wild type CCDC6 protein (Fig. 6C, Additional File 11: Figure S8).

In addition, the suppression of the regulatory component PP4R3α hampered the rescue of the CCDC6 wild type or CCDC6T427A mutant plasmids overexpression, as measured by cell survival experiments, upon olaparib treatment, by blocking the CCDC6-PP4 interaction through the FxxP motif (Fig. 6D, E).

According to our findings, the PP4 complex activity, which is made up of the catalytic subunit PP4c and the regulatory subunit 3α, (PP4R3α,), is necessary for the CCDC6-dependent BRCA-ness status.

## Discussion

The most deadly gynecological cancer and one of the leading causes of cancer death in women is epithelial ovarian carcinoma [52]. Approximately 70% of all epithelial ovarian cancers are HGSOC, while between 85 and 90% of all ovarian malignancies are epithelial in origin [53]. Different histological entities (such as high-grade serous, endometrioid, mucinous, clear cell, and low-grade serous) with different biological, molecular, and clinical features make up epithelial ovarian cancer, which is not a single disease but is treated using a similar strategy that entails debulking surgery followed by adjuvant chemotherapy. The majority of women with advanced-stage epithelial ovarian cancer, nevertheless, will also relapse, necessitating further therapy [18].

Due to genetic and epigenetic changes in HR pathway genes, over 50% of ovarian tumours show poor DNA repair by homologous recombination. The effectiveness of platinum medicines in treating epithelial ovarian cancer and the development of PARP inhibitors, which exhibit synthetic lethality when administered to HR-deficient cells, both demonstrate that defective HR is a relevant therapeutic target in this cancer. Furthermore, the PARP inhibitors' extraordinary activity in cellular systems lacking HR repair has been demonstrated in preclinical investigations by a synthetic lethality rationale, and this activity has been confirmed in clinical trials in *BRCA1/2* mutation carriers with ovarian carcinomas [7].

The PARP inhibitors olaparib, niraparib, and rucaparib have been authorized and introduced as maintenance treatment in patients with ovarian cancer who have experienced a platinum-sensitive recurrence since their initial admission for clinical usage in 2014 [9, 18, 54–57]. Unfortunately, the *BRCA1/2* mutations or the presence of an HR deficit affected the reported medication response. However, new studies imply that these substances are similarly active in tumours harbouring wild type *BRCA1/2* [45]. The discovery of new biomarkers is therefore highly valued in order to increase the applicability of PARP inhibitors and improve the quality of response [52]. Here, we provide preclinical data demonstrating an association between olaparib sensitivity and the CCDC6 gene product depletion in ovarian cancer, indicating CCDC6 as one of these biomarkers.

Due to gene translocations, somatic mutations, and altered protein levels, the tumour suppressor protein CCDC6 is known to lose its functional properties in several tumour types. When exposed to DNA damage, CCDC6 moves to the nucleus where it interacts with the HR machinery by binding to the protein phosphatase PP4c and controlling the phosphorylation of H2AX [28]. Notably, CCDC6 protein levels and functions are carefully regulated by the balance between the E3 ubiquitin ligase FBXW7 and the de-ubiquitinase USP7 activities [30]. Here, we demonstrate how synthetic lethality is conferred to clinically relevant PARPi in a range of HGSOC cell lines by CCDC6 downregulation, which may be accomplished either genetically, by transfection of short hairpin RNA, or pharmacologically, by using a pharmacological USP7 inhibitor. Interestingly, we discovered that CCDC6 attenuation increased the sensitivity to olaparib and acts synergistically with cisplatin in primary (PEO4) and newly generated (OVCAR3 olaR and OV-90 olaR) olaparib-resistant cells of HGSOC. This was accomplished by affecting the formation of H2AX foci and the repair of DNA DSBs by HR. Surprisingly, CCDC6 downregulation predicted susceptibility to many classes of PARP inhibitors, regardless of each compound's unique capacity to trap or inhibit PARP. Notably, reports of the effectiveness of veliparib and talazoparib in patients with *BRCA* WT, *BRCA* mutation-tested patients, or patients with HR DNA repair abnormalities under investigation, indicate novel additional pathways providing PARPi sensitivity [9, 57].

As a novel treatment strategy for PARPi-resistant HGSOCs, CCDC6 loss of function confers resistance to PARG inhibitor [24]. Comparing the PARPi response to the varied sensitivity to PARG inhibitors in cells with CCDC6 attenuation, it is possible to infer that the two inhibitors have separate mechanisms of action. The significant effects of PARPi, however, are replication stress, replication fork collapse, and double strand breaks [58], all of which can be repaired by HR repair. In this case, CCDC6 depletion might worsen DNA damage by preventing HR repair. By slowing down replication forks, accumulating inverted forks and ssDNA gaps, and inhibiting the RECQ1 helicase, PARG depletion or inhibition, on the other hand, results in cell cycle stalling in the S/G2 phase [24–26, 59–62].

**Fig. 7:**
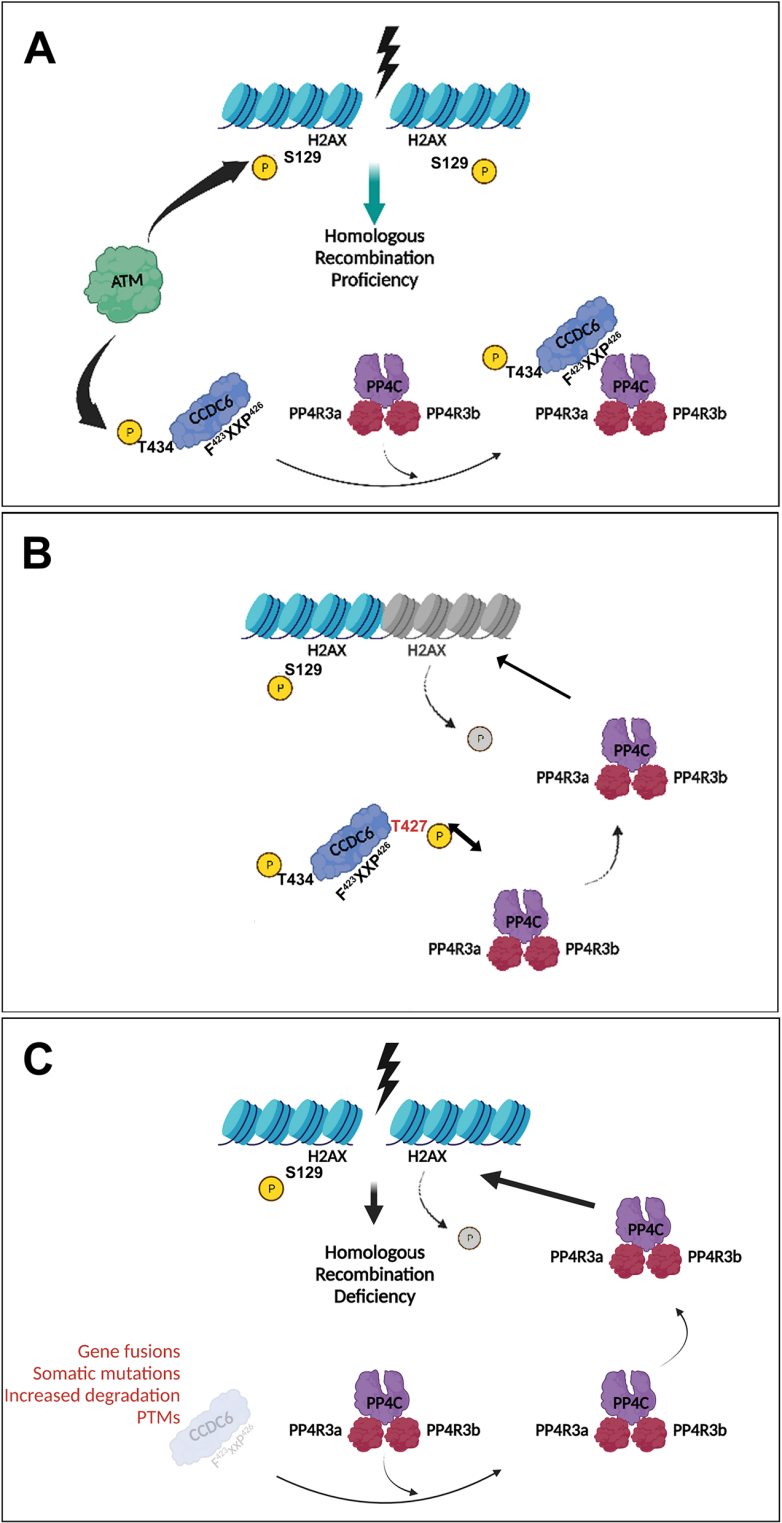
CCDC6 is a functional regulator of the PP4c Phosphatase activity. **A** The ATM kinase phosphorylates histone H2AX at S129 in the presence of DNA Double Strand Breaks (DSBs) to initiate homologous recombination DNA repair. The protein phosphatase PP4c, which is complexed with the PP43α/β regulatory subunits, dephosphorylates histone H2AX at the completion of the repair process, allowing the cell cycle to continue. The ATM kinase also phosphorylates the CCDC6 protein at residue Threonine 434 in response to DNA damage, which adversely controls the phosphatase function of the PP4c holoenzyme via the F423xxP426 motif, retaining the phosphorylated histone H2AX activity. **B** The phosphorylation of Threonine 427, which flanks by the CCDC6-PP4c interaction motif, inhibits CCDC6 from binding to PP4, allowing its phosphatase activity on γH2AX to be released, resulting in HR deficiency and, as a result, PARPi sensitivity. **C** This effect also occurs when PP4c is released in response to the physical or functional loss of CCDC6, which might be caused by a variety of processes (e.g., CCDC6 truncation after gene fusion, somatic mutations, or accelerated degradation due to post translational alterations, etc.)

Here, we report that CCDC6 phosphorylation at position T427 altered the activation of H2AX and the repair of DNA DSBs by HR, simulating the loss of CCDC6 or its functional impairment, by inhibiting the connection between CCDC6, the PP4c phosphatase, and the PP4R3 regulatory subunits [29]. This data favours a molecular mechanism that can account for the sensitivity of PARP inhibitors in the absence of CCDC6 activity. The conserved PP4 holoenzyme's consensus-binding motif (FxxP) for CCDC6 has recently been characterized, and it has been shown to give PP4 substrate specificity [29]. The rescue experiments carried out with the WT or unphosphorylated point mutant (T427A) CCDC6 isoforms, which are still able to interact and control the phosphatase activity, provide significant support for this observation (Fig. 7). Surprisingly, we also saw a high correlation between the regulatory component PP4R3 and the PARPi sensitivity. In fact, PP4R3 deletion increased the sensitivity to PARPi while also altering the rescue effect of transient transfections of either the WT or T427A mutant CCDC6 isoforms. These findings clearly suggest that the BRCAness phenotype connected to the lack of CCDC6 function depends on the CCDC6-PP4c complex activity. The PP4c functional role and its involvement in multiple aspects of cellular physiology are emerging [63]. Nevertheless, the CCDC6 ability to interact and modulate the activity of the PP4c complex resulted in relevant responses to genotoxic stress and oxidative stress, highlighting the CCDC6 gene product as a stress key player [36, 63–65].

We detected substantial levels of CCDC6 protein expression in olaparib-resistant cell cultures, and most crucially, in olaparib-resistant PDX, as shown by immunoblot or immunohistochemistry (Fig. 5, Additional File 10: Figure S7 D-G). We hypothesized, based on in vitro evidence, that the intensity of the CCDC6 staining could nonetheless predict the response to PARPi, as described in other tumour models [36–41]. However, the CCDC6 staining of PDX cores did not appear sufficient to distinguish a primary sensitive from a resistant tumour or to predict the cisplatin sensitivity. Relapse-resistant patient-derived xenografts may one day serve as an effective in vivo model to study how various medication combinations affect a patient's response to therapy. The ovarian PDX may then become susceptible to PARP inhibitors that can work in synergy with cisplatin after CCDC6 downregulation is induced by USP7 inhibitors [66]. Taking into account the *BRCA1/2* mutational status of the xenografts, this approach may offer a potential entry point to address the chemoresistance problems associated with single-agent treatment.

Platinum resistance in ovarian cancer remains an unmet clinical need and a significant challenge, so it is critical to find molecular biomarkers that can predict how patients will respond to platinum-based therapy. This will enable patient stratification and the development of alternative therapeutic approaches. In addition, soon, it will be possible to prevent or detect tumor relapses sooner thanks to the ability to follow gene product modifications in cells during liquid biopsy [67, 68].

It's interesting to note that CCDC6 has been immunostained on a collection of primary ovarian tissues (*N* = 251, organized in a Tissue MicroArray (TMA)). Unfortunately, most of the cores showed intense staining of the CCDC6 protein (median H-SCORE was 64.2 (IQR 28.8; 97.2); mean H-SCORE was 67.5 (sd 47.1). (Additional File 12: Figure S9). In addition, nearly 10% of the cores of our research cases (30/251) also had poor expression levels of the CCDC6 protein as well. However, according to the MITO-2 randomised phase III study, all the patient-derived specimens were collected from women who underwent first-line therapy and were not known to have detrimental *BRCA1* or *BRCA2* mutations (Additional File 2–3: Tables S2 and S3) [33, 69].

We are currently conducting biochemical studies and analysing additional ovarian cancer samples, as well as patient-derived xenografts, enriched for *BRCA1/2* mutants, to understand whether the *BRCA1/2* mutational status is associated with or determines the CCDC6 increased turnover. This is because in the *BRCA2* mutant PEO1 ovarian cancer cells only a barely detection of CCDC6 protein was observed at immunoblot, likely due to an enhanced CCDC6 proteolysis. This finding may have therapeutic significance since it will promote the use of CCDC6 as a biomarker for early-individualized therapy employing targeted treatments. In addition, Patients-Derived Organoids, or PDOs, have also recently been thought to be practical for examining HGSOC's susceptibility to and resistance to PARPi [70, 71].

## Conclusion

As a result of our research, we can say that the disruption of the CCDC6-PP4c axis is related to the phenotype of BRCAness and susceptibility to PARP inhibitors. This suggests that targeting the PP4 complex components may be a proper treatment strategy. Furthermore, our findings point to the possibility of directly or indirectly targeting CCDC6 in order to address PARPi-resistance and to restore the cisplatin sensitivity in HGSOCs.

A routine determination of the CCDC6 protein levels by IHC, along with *BRCA1/2* genotyping by next-generation sequencing, may be advantageous for predicting PARPi sensitivity and customizing HGSOC therapeutic decisions. This is because there are low expression levels of CCDC6 protein have been found in nearly 10% of primary ovarian specimens (30/251) and the increased turnover of CCDC6 may depend on *BRCA2* deleterious mutations.

## Supplementary Information

**Additional file 1: Table S1.** Genetic characteristics of the HGSOC cells.

**Additional file 2: Figure S1.** Schematic representation of the DR-GFP reporter plasmid. The reporter plasmid DR**-**GFP consists of two mutated GFP cassettes. The GFP cassette at the top contains a binding site for I-SceI restriction enzyme, whose expression causes a Double Strand Break (DSB). In Homologous Recombination (HR) proficient cells, the GFP available in the cassette on the bottom serves as template for the repair of I**-**SceI damaged GFP sequence, restoring the GFP expression.

**Additional file 3: Table S2.** H-scores of the CCDC6 immunohistochemical staining.

**Additional file 4: Table S3.** Study population of ovarian cancers tested for CCDC6 protein expression.

**Additional file 5: Figure S2.** Chemical inhibition of the de-ubiquitinase USP7 by P5091 affects CCDC6 stability. Immunoblot analysis of CCDC6 in Kuramochi, (A) OVCAR3 (B) and OV-90 (C) cells silenced for CCDC6 by transfection with short hairpin (ShCCDC6) or control, (ShCTRL). Tubulin served as a loading control. Kuramochi (D), OVCAR3 (E) and OV-90 (F) ovarian cancer cells were pre-treated either with vehicle or P5091 [5μM] for 4 hours, followed by the addition of cycloheximide (CHX) at 50 μg/ml for the indicated times. Total protein lysates were subjected to immunoblot analysis using anti-CCDC6 or anti**-**PCNA antibodies. Densitometric analyses have been performed by Image J Software. The histograms represent the relative protein levels of CCDC6 against PCNA and expressed as relative intensity compared to untreated. Error bars indicate the measurement of the standard error mean. Statistical significance was verified by 2**-**tailed Student's t-test (* *p* <0.05; ** *p* <0.01 and *** *p* <0.001). (G, H) Immunoblot analysis of USP7, PARG, PARP1, CCDC6 and pan-ADP-Ribose in human Kuramochi, OVCAR3 and OV-90 ovarian cancer cell lines. Anti-Tubulin is shown as loading control. The different CCDC6 protein mobility on SDS-PAGE could be ascribed to cell cycle-dependent CCDC6 post-translational modifications (PTMs), as reported [30].

**Additional file 6: Figure S3.** In CCDC6-silenced Kuramochi cells (ShCCDC6), the γH2AX foci formation was rescued by CCDC6 exogenous expression upon Myc CCDC6 transient transfection (Myc CCDC6) vs empty vector (EV) as control. (A) Immunofluorescence images showing γH2AX nuclear foci formation in CCDC6-silenced Kuramochi cells, treated with Olaparib [1μM] or PARGi [1μM] for 48 hours and transfected with control (EV) or Myc CCDC6 expression vector. Scale bar 50μm. (B) Graphs represent the percentage of cells with more than 15 foci. Error bars indicate the standard error mean derived from three independent experiments. Statistical significance was verified by 2-tailed Student's t-test (* *p* <0.05; ** *p* <0.01 and *** *p* <0.001). (C) The efficacy of CCDC6 silencing and the expression of Myc CCDC6 were assessed at Western Blot by the anti-CCDC6 and anti-Myc antibodies. Anti-Tubulin immunoblots are served as a loading control.

**Additional file 7: Figure S4**. In CCDC6-silenced OVCAR3 cells (ShCCDC6), the γH2AX foci formation was rescued by CCDC6 exogenous expression upon Myc CCDC6 transient transfection (Myc CCDC6) vs empty vector (EV) as control. (A) Immunofluorescence images showing γH2AX nuclear foci formation in CCDC6-silenced OVCAR3 cells, treated with olaparib [1μM] or PARGi [1μM] for 48 hours and transfected with control (EV) or Myc CCDC6 expression vector. Scale bar 50μm. (B) Graphs represent the percentage of cells with more than 15 foci. Error bars indicate the standard error mean derived from three independent experiments. Statistical significance was verified by 2-tailed Student's t-test (* *p* <0.05; ** *p* <0.01 and *** *p* <0.001). (C) The efficacy of CCDC6 silencing and the expression of Myc CCDC6 were assessed at Western Blot by the anti-CCDC6 and anti-Myc antibodies. Anti-Tubulin immunoblots are served as a loading control.

**Additional file 8: Figure S5.** In CCDC6-silenced OV-90 cells (ShCCDC6), the γH2AX foci formation was rescued by CCDC6 exogenous expression upon Myc CCDC6 transient transfection (Myc CCDC6) vs empty vector (EV) as control. (A) Immunofluorescence images showing γH2AX nuclear foci formation in CCDC6-silenced OV-90 cells, treated with olaparib [1μM] or PARGi [1μM] for 48 hours and transfected with control (EV) or Myc CCDC6 expression vector. Scale bar 50μm. (B) Graphs represent the percentage of cells with more than 15 foci. Error bars indicate the standard error mean derived from three independent experiments. Statistical significance was verified by 2-tailed Student's t-test (* *p* <0.05; ** *p* <0.01 and *** *p* <0.001). (C) The efficacy of CCDC6 silencing and the expression of myc-CCDC6 were assessed at Western Blot by the anti-CCDC6 and anti-myc antibodies. Anti-tubulin immunoblots are served as a loading control.

**Additional file 9: Figure S6.** CCDC6 genetic depletion by short hairpin RNA (ShCCDC6) improved Olaparib sensitivity in HGSOC cells. (A, D, G) Kuramochi, OVCAR3 and OV-90 cells, transfected with ShCCDC6, or ShCTRL were treated with olaparib or PARGi at different doses for 144 hours: the drugs sensitivity was determined by a modified 3**-**(4,5**-**dimethylthiazole**-**2**-**yl)**-**2**-**5**-**diphenyltetrazolium bromide assay, CellTiter 96 Aqueous One Solution assay (Promega) and expressed as 50% inhibitory concentration (IC50) values. (B, E, H) In P5091-treated CCDC6-depleted cells, the sensitive phenotypes were rescued by CCDC6 exogenous expression upon Myc CCDC6 transient transfection (CCDC6+) vs empty vector (EV) as control. The drugs sensitivity was determined as in A, D, G. (C, F, I) The efficacy of CCDC6 silencing or of Myc CCDC6 expression were assessed at Western Blot by the anti-CCDC6 or anti-Myc antibodies. Anti-Tubulin immunoblots are shown as loading control

**Additional file 10: Figure S7. **CCDC6 deficiency or chemical downregulation, by impairing the homology-directed repair in PEO1 and PEO4 ovarian cancer cells, increases PARP inhibitors sensitivity. (A) The Myc CCDC6 expression in CCDC6 null PEO1 cells, and the efficacy of CCDC6 silencing, upon ShCCDC6 transient transfection in CCDC6 proficient PEO4 cells, were assessed by anti-Myc and anti-CCDC6 antibodies at Western Blot. (B) In PEO1 cells, transfected with DR-GFP alone, HA-ISceI and both HA-ISceI and CCDC6 wild type and (C) in PEO4 cells, pre-treated either with vehicle or P5091 [2.5μM] for 4 hours and transfected with the above-mentioned plasmids, the percentages of GFP positive cells, compared to controls, were plotted as histograms, representative of the mean of three independent experiments. Error bars indicate the measurement of the standard error mean. Statistical significance was verified by 2**-**tailed Student's t-test (* *p* <0.05; ** *p* <0.01 and *** *p* <0.001). The Myc CCDC6 and HA-ISceI protein expression were assessed respectively by anti**-**Myc and anti**-**HA antibodies, at Western Blot. Anti-Tubulin immunoblots are shown as loading control. (D) CCDC6 protein expression was assayed, following cell block procedure, by immunohistochemistry on the ovarian cancer cell lines (a, b) OV90, parental and olaR, (c,d) OVCAR3, parental and olaR, (e, f) PEO1 transiently expressing Empty Vector (EV) or CCDC6 wild type (Myc CCDC6), respectively, (g, h) PEO4 transfected with ShCTRL and ShCCDC6, respectively. An automatic count of positive cells was performed on digital slides with QuPath image analysis software, and results are shown in the histograms (E). Statistical significance was verified by chi-square test (* *p* <0.05; ** *p* <0.01 and *** *p* <0.001). (F) CCDC6 protein expression was also evaluated at Western Blot, as indicated. Anti**-**Tubulin immunoblot is shown as loading control. (G) Densitometric analysis have been performed by Image J Software. The histograms represent the relative protein levels of CCDC6 normalised to Tubulin and expressed as relative intensity compared to untreated cells. Error bars indicate the standard error mean derived from three independent experiments. Statistical significance was verified by 2**-**tailed Student's t**-**test (* *p* <0.05; ** *p* <0.01 and *** *p* <0.001).

**Additional file 11: Figure S8. **Densitometric analysis have been performed by Image J Software. The histograms represent the relative protein levels of γH2AX normalised to total H2AX and expressed as relative intensity compared to control. Error bars indicate the measurement of the standard error mean. Statistical significance was verified by 2-tailed Student's t-test (* *p* <0.05; ** *p* <0.01 and *** *p* <0.001).

**Additional file 12: Figure S9. **CCDC6 IHC expression in ovary tumours. Three representative samples of “high” CCDC6 expression (#1, #2, #3) and three representative cases of “low” CCDC6 expression (#4, #5, #6) are shown. For each sample, 10x, 20x, and 40x magnification fields are shown along with the whole TMA core of the digitally scanned glass slide.

## Data Availability

All data generated or analysed during this study are included in this published article [and its supplementary information files].

## Abbreviations

HGSOC: High-grade serous ovarian cancers
DDR: DNA damage repair response
DSBs: Double strand breaks
HR: Homologous recombination
PARP: Poly(ADP-ribose) polymerase
PARPi: PARP inhibitors
PARylation: Poly(ADP-ribosyl)ation
PARG: Poly(ADP-ribose) glycohydrolase
PARGi: PARG inhibitor (PDD00017273)
HPF1: Histone PARylation Factor 1
PDX: Patient derived xenografts (PDXs)
MNHOC: Mario Negri Human Ovarian Cancer
FFPE: Formalin fixed paraffin embedded
CTRL: Control
WT: Wild type
CI: Combination index
EV: Empty vector
TMA: Tissue MicroArray
IHC: Immunohistochemistry
PDO: Patients-Derived Organoids
