# Supplementary figures and images for: The disruption of the CCDC6 – PP4 axis induces a BRCAness like phenotype and sensitivity to PARP inhibitors in high-grade serous ovarian carcinoma
Source: J Exp Clin Cancer Res. 2022 Aug 13;41:245. doi: 10.1186/s13046-022-02459-2 (PMC9375931; doi:10.1186/s13046-022-02459-2)

Table S1

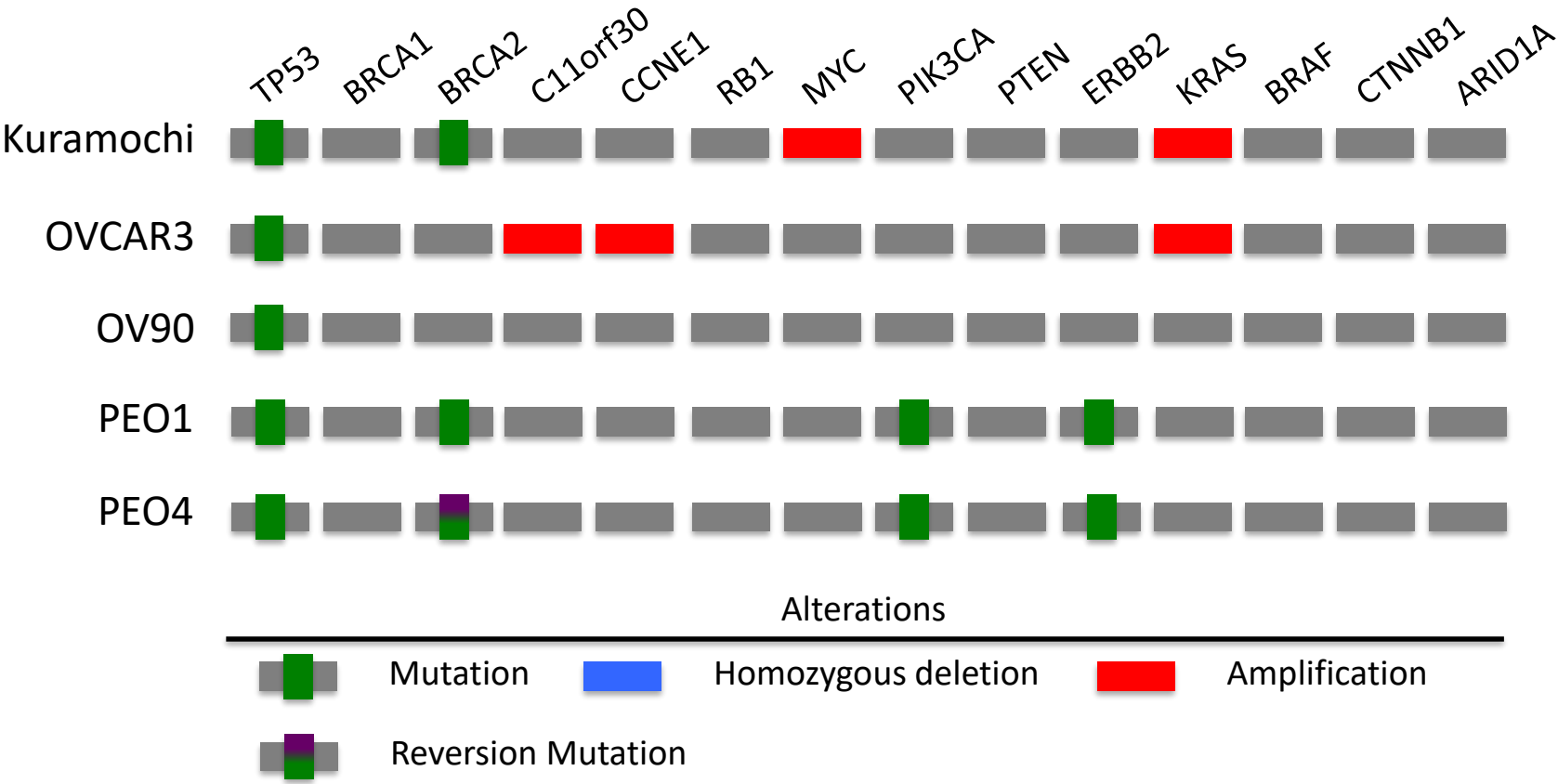

Supplement: Supplementary file 1 — Additional file 1: Table S1. Genetic characteristics of the HGSOC cells. [file 13046_2022_2459_MOESM1_ESM.pdf]

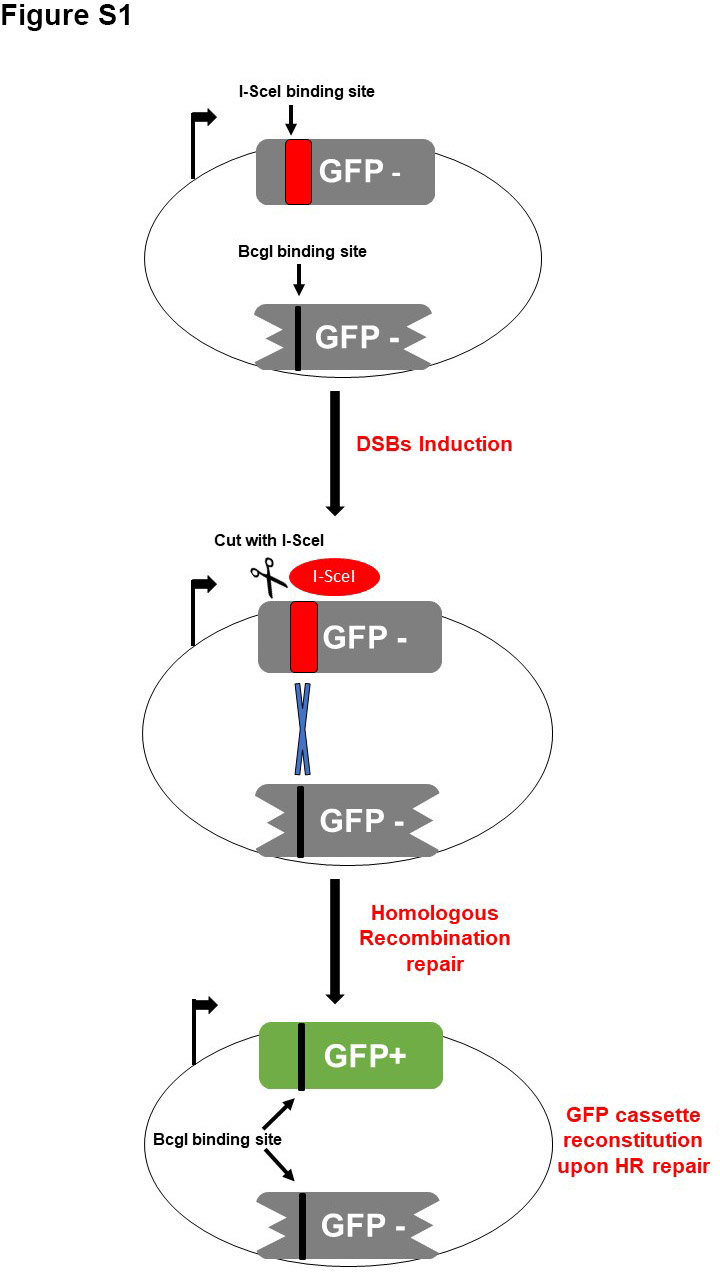

Supplement: Supplementary file 2 — Additional file 2: Figure S1. Schematic representation of the DR-GFP reporter plasmid. The reporter plasmid DR-GFP consists of two mutated GFP cassettes. The GFP cassette at the top contains a binding site for I-SceI restriction enzyme, whose expression causes a Double Strand Break (DSB). In Homologous Recombination (HR) proficient cells, the GFP available in the cassette on the bottom serves as template for the repair of I-SceI damaged GFP sequence, restoring the GFP expression. [file 13046_2022_2459_MOESM2_ESM.jpg]

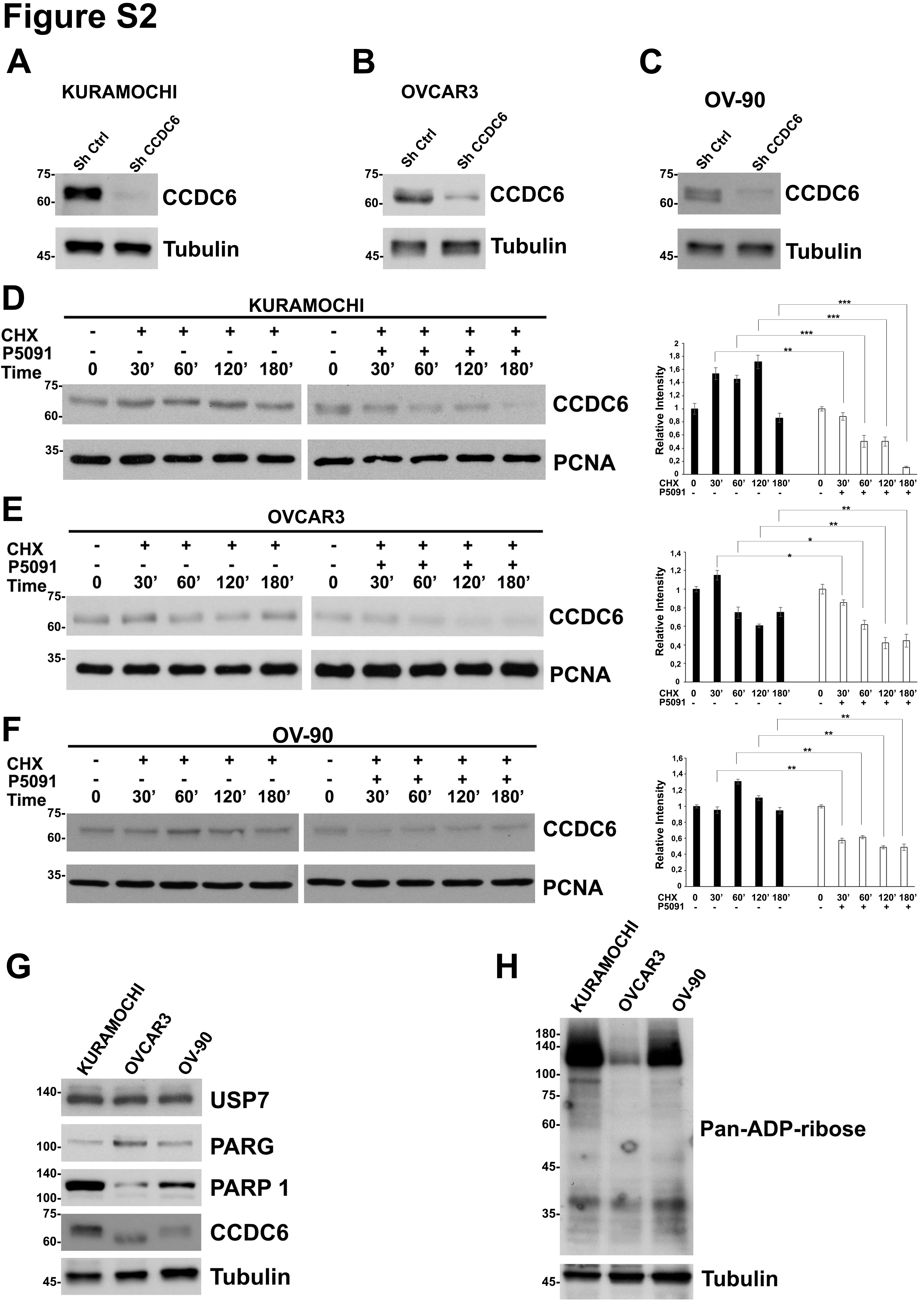

Supplement: Supplementary file 5 — Additional file 5: Figure S2. Chemical inhibition of the de-ubiquitinase USP7 by P5091 affects CCDC6 stability. Immunoblot analysis of CCDC6 in Kuramochi, (A) OVCAR3 (B) and OV-90 (C) cells silenced for CCDC6 by transfection with short hairpin (ShCCDC6) or control, (ShCTRL). Tubulin served as a loading control. Kuramochi (D), OVCAR3 (E) and OV-90 (F) ovarian cancer cells were pre-treated either with vehicle or P5091 [5μM] for 4 hours, followed by the addition of cycloheximide (CHX) at 50 μg/ml for the indicated times. Total protein lysates were subjected to immunoblot analysis using anti-CCDC6 or anti-PCNA antibodies. Densitometric analyses have been performed by Image J Software. The histograms represent the relative protein levels of CCDC6 against PCNA and expressed as relative intensity compared to untreated. Error bars indicate the measurement of the standard error mean. Statistical significance was verified by 2-tailed Student's t-test (* p <0.05; ** p <0.01 and *** p <0.001). (G, H) Immunoblot analysis of USP7, PARG, PARP1, CCDC6 and pan-ADP-Ribose in human Kuramochi, OVCAR3 and OV-90 ovarian cancer cell lines. Anti-Tubulin is shown as loading control. The different CCDC6 protein mobility on SDS-PAGE could be ascribed to cell cycle-dependent CCDC6 post-translational modifications (PTMs), as reported [30]. [file 13046_2022_2459_MOESM5_ESM.jpg]

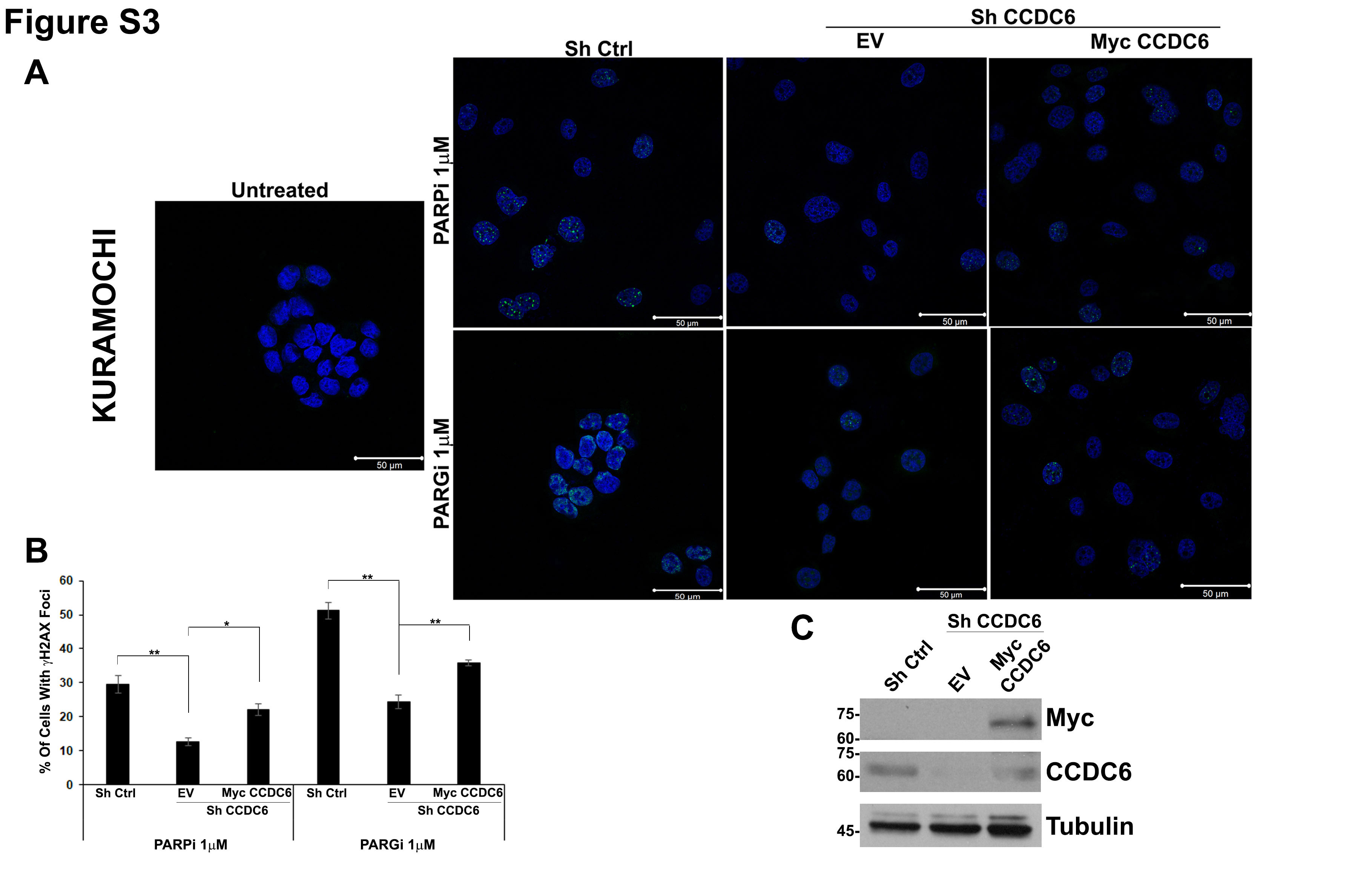

Supplement: Supplementary file 6 — Additional file 6: Figure S3. In CCDC6-silenced Kuramochi cells (ShCCDC6), the γH2AX foci formation was rescued by CCDC6 exogenous expression upon Myc CCDC6 transient transfection (Myc CCDC6) vs empty vector (EV) as control. (A) Immunofluorescence images showing γH2AX nuclear foci formation in CCDC6-silenced Kuramochi cells, treated with Olaparib [1μM] or PARGi [1μM] for 48 hours and transfected with control (EV) or Myc CCDC6 expression vector. Scale bar 50μm. (B) Graphs represent the percentage of cells with more than 15 foci. Error bars indicate the standard error mean derived from three independent experiments. Statistical significance was verified by 2-tailed Student's t-test (* p <0.05; ** p <0.01 and *** p <0.001). (C) The efficacy of CCDC6 silencing and the expression of Myc CCDC6 were assessed at Western Blot by the anti-CCDC6 and anti-Myc antibodies. Anti-Tubulin immunoblots are served as a loading control. [file 13046_2022_2459_MOESM6_ESM.jpg]

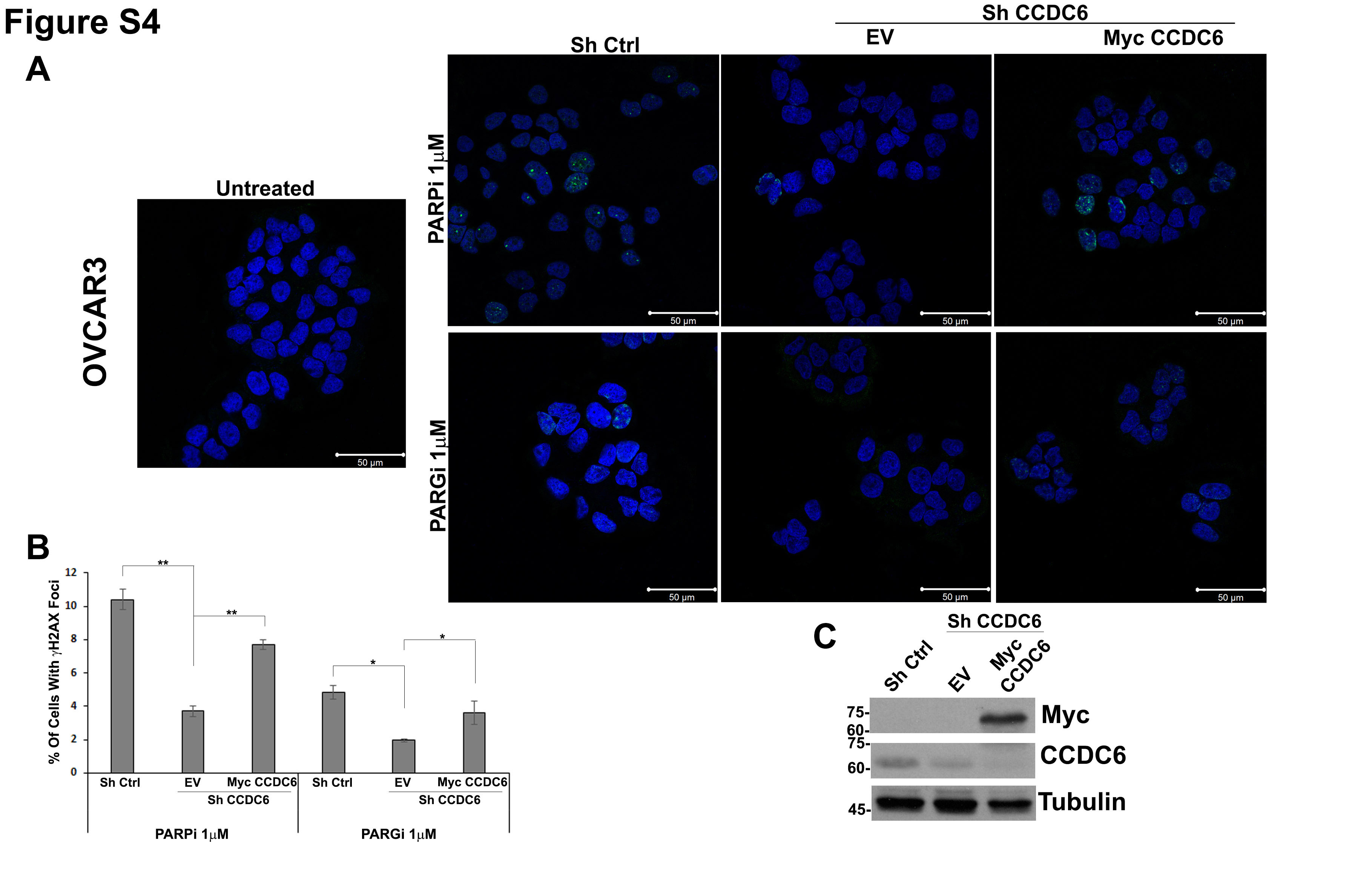

Supplement: Supplementary file 7 — Additional file 7: Figure S4. In CCDC6-silenced OVCAR3 cells (ShCCDC6), the γH2AX foci formation was rescued by CCDC6 exogenous expression upon Myc CCDC6 transient transfection (Myc CCDC6) vs empty vector (EV) as control. (A) Immunofluorescence images showing γH2AX nuclear foci formation in CCDC6-silenced OVCAR3 cells, treated with olaparib [1μM] or PARGi [1μM] for 48 hours and transfected with control (EV) or Myc CCDC6 expression vector. Scale bar 50μm. (B) Graphs represent the percentage of cells with more than 15 foci. Error bars indicate the standard error mean derived from three independent experiments. Statistical significance was verified by 2-tailed Student's t-test (* p <0.05; ** p <0.01 and *** p <0.001). (C) The efficacy of CCDC6 silencing and the expression of Myc CCDC6 were assessed at Western Blot by the anti-CCDC6 and anti-Myc antibodies. Anti-Tubulin immunoblots are served as a loading control. [file 13046_2022_2459_MOESM7_ESM.jpg]

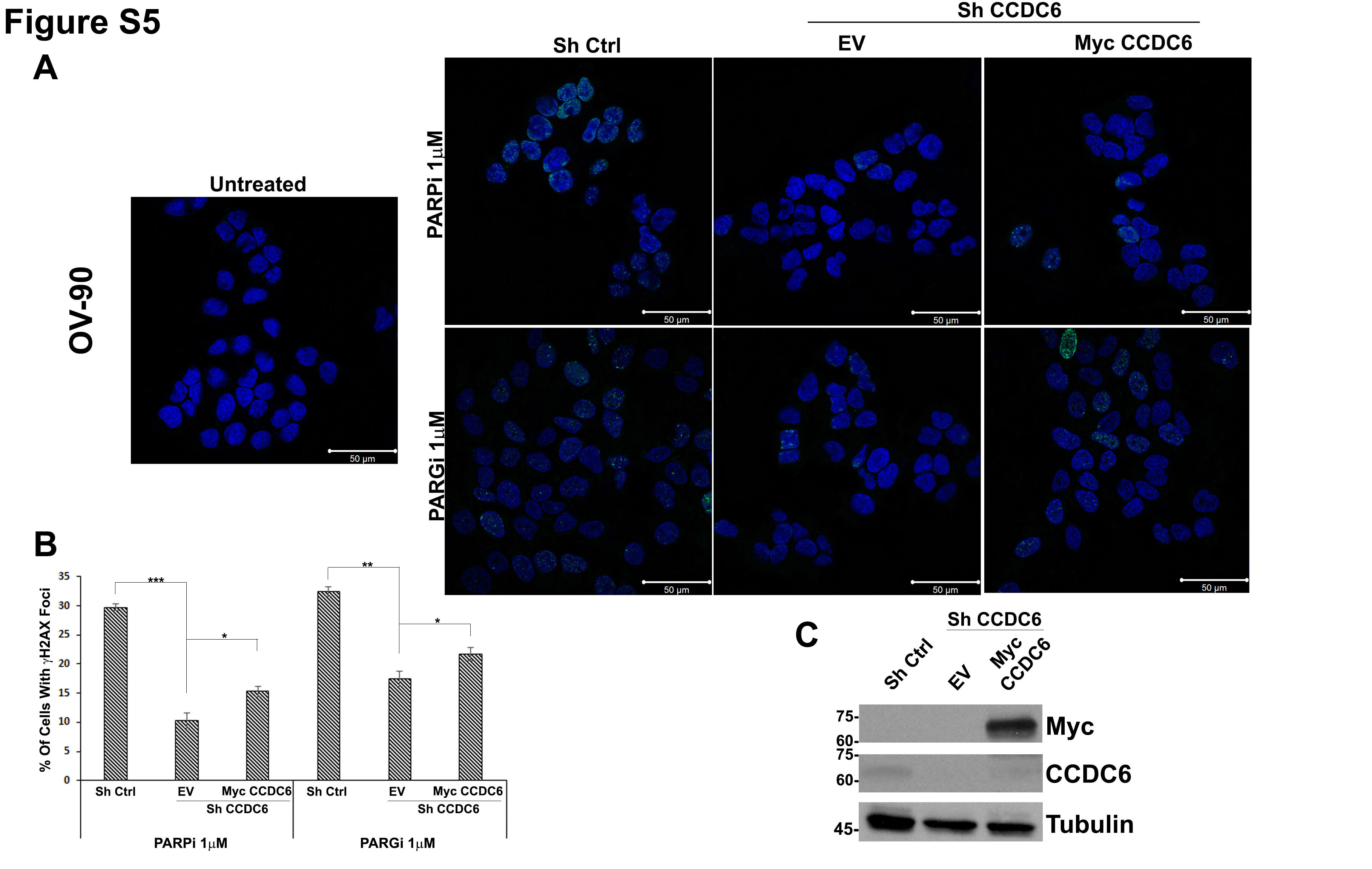

Supplement: Supplementary file 8 — Additional file 8: Figure S5. In CCDC6-silenced OV-90 cells (ShCCDC6), the γH2AX foci formation was rescued by CCDC6 exogenous expression upon Myc CCDC6 transient transfection (Myc CCDC6) vs empty vector (EV) as control. (A) Immunofluorescence images showing γH2AX nuclear foci formation in CCDC6-silenced OV-90 cells, treated with olaparib [1μM] or PARGi [1μM] for 48 hours and transfected with control (EV) or Myc CCDC6 expression vector. Scale bar 50μm. (B) Graphs represent the percentage of cells with more than 15 foci. Error bars indicate the standard error mean derived from three independent experiments. Statistical significance was verified by 2-tailed Student's t-test (* p <0.05; ** p <0.01 and *** p <0.001). (C) The efficacy of CCDC6 silencing and the expression of myc-CCDC6 were assessed at Western Blot by the anti-CCDC6 and anti-myc antibodies. Anti-tubulin immunoblots are served as a loading control. [file 13046_2022_2459_MOESM8_ESM.jpg]

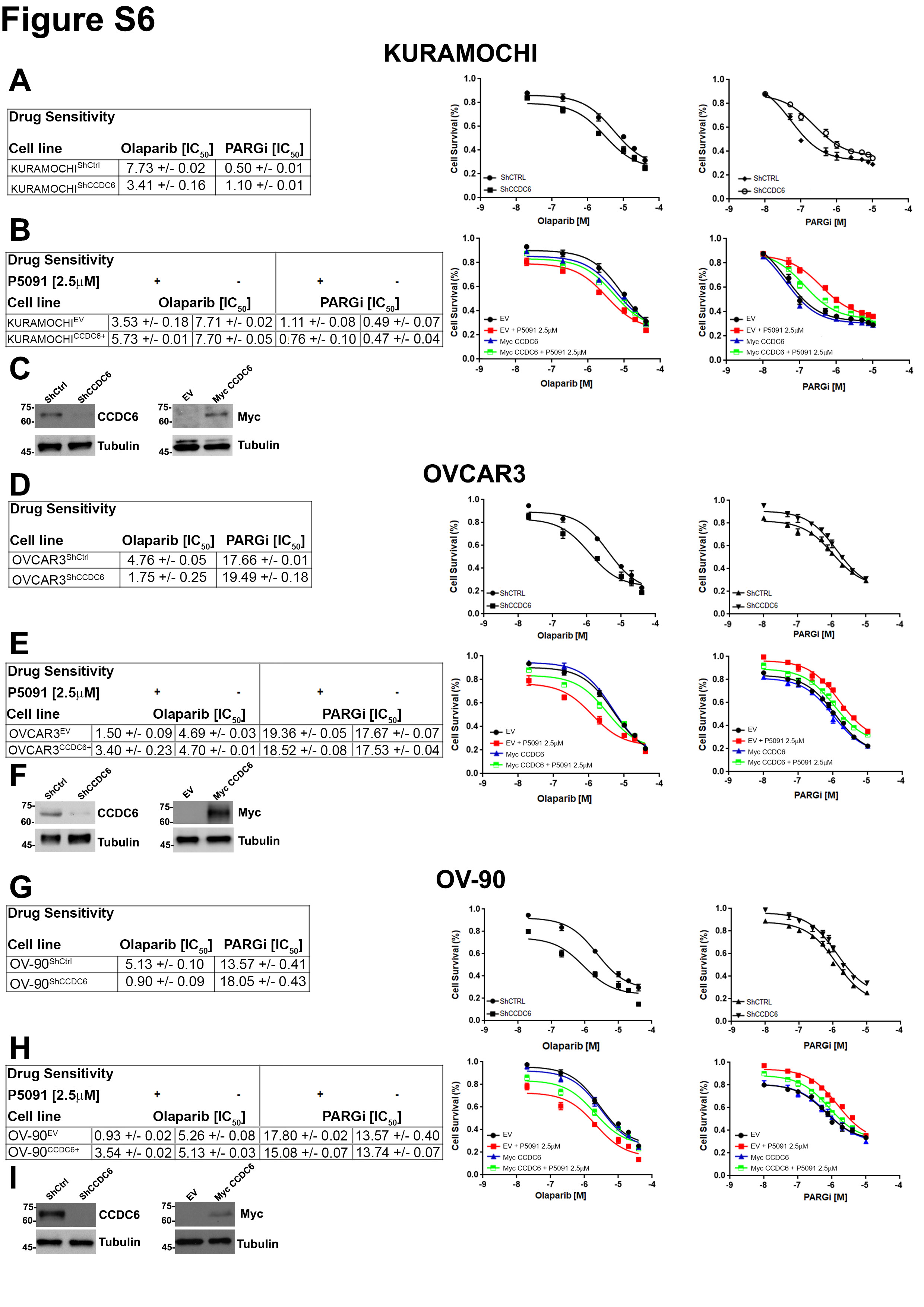

Supplement: Supplementary file 9 — Additional file 9: Figure S6. CCDC6 genetic depletion by short hairpin RNA (ShCCDC6) improved Olaparib sensitivity in HGSOC cells. (A, D, G) Kuramochi, OVCAR3 and OV-90 cells, transfected with ShCCDC6, or ShCTRL were treated with olaparib or PARGi at different doses for 144 hours: the drugs sensitivity was determined by a modified 3-(4,5-dimethylthiazole-2-yl)-2-5-diphenyltetrazolium bromide assay, CellTiter 96 Aqueous One Solution assay (Promega) and expressed as 50% inhibitory concentration (IC50) values. (B, E, H) In P5091-treated CCDC6-depleted cells, the sensitive phenotypes were rescued by CCDC6 exogenous expression upon Myc CCDC6 transient transfection (CCDC6+) vs empty vector (EV) as control. The drugs sensitivity was determined as in A, D, G. (C, F, I) The efficacy of CCDC6 silencing or of Myc CCDC6 expression were assessed at Western Blot by the anti-CCDC6 or anti-Myc antibodies. Anti-Tubulin immunoblots are shown as loading control [file 13046_2022_2459_MOESM9_ESM.jpg]

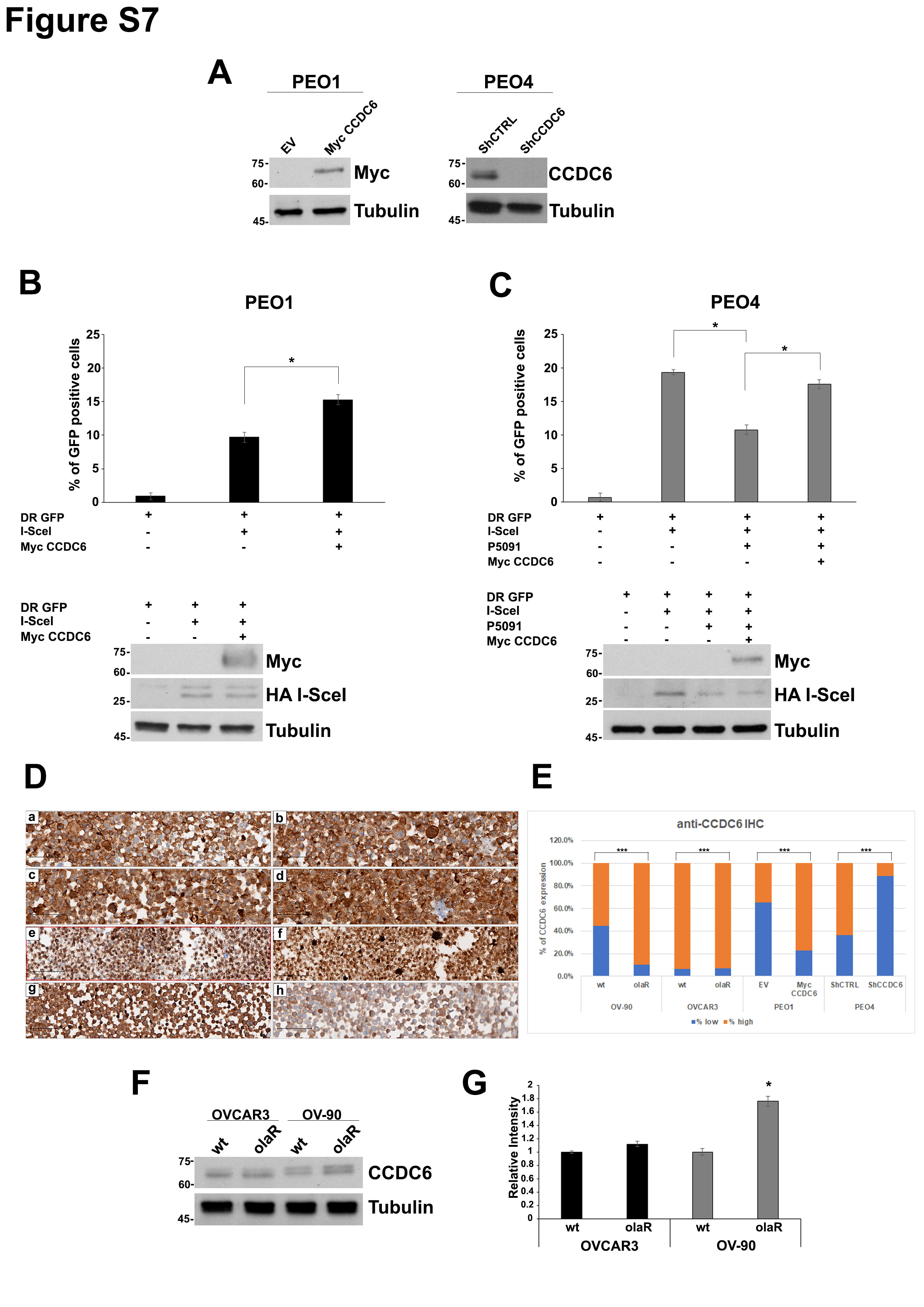

Supplement: Supplementary file 10 — Additional file 10: Figure S7. CCDC6 deficiency or chemical downregulation, by impairing the homology-directed repair in PEO1 and PEO4 ovarian cancer cells, increases PARP inhibitors sensitivity. (A) The Myc CCDC6 expression in CCDC6 null PEO1 cells, and the efficacy of CCDC6 silencing, upon ShCCDC6 transient transfection in CCDC6 proficient PEO4 cells, were assessed by anti-Myc and anti-CCDC6 antibodies at Western Blot. (B) In PEO1 cells, transfected with DR-GFP alone, HA-ISceI and both HA-ISceI and CCDC6 wild type and (C) in PEO4 cells, pre-treated either with vehicle or P5091 [2.5μM] for 4 hours and transfected with the above-mentioned plasmids, the percentages of GFP positive cells, compared to controls, were plotted as histograms, representative of the mean of three independent experiments. Error bars indicate the measurement of the standard error mean. Statistical significance was verified by 2-tailed Student's t-test (* p <0.05; ** p <0.01 and *** p <0.001). The Myc CCDC6 and HA-ISceI protein expression were assessed respectively by anti-Myc and anti-HA antibodies, at Western Blot. Anti-Tubulin immunoblots are shown as loading control. (D) CCDC6 protein expression was assayed, following cell block procedure, by immunohistochemistry on the ovarian cancer cell lines (a, b) OV90, parental and olaR, (c,d) OVCAR3, parental and olaR, (e, f) PEO1 transiently expressing Empty Vector (EV) or CCDC6 wild type (Myc CCDC6), respectively, (g, h) PEO4 transfected with ShCTRL and ShCCDC6, respectively. An automatic count of positive cells was performed on digital slides with QuPath image analysis software, and results are shown in the histograms (E). Statistical significance was verified by chi-square test (* p <0.05; ** p <0.01 and *** p <0.001). (F) CCDC6 protein expression was also evaluated at Western Blot, as indicated. Anti-Tubulin immunoblot is shown as loading control. (G) Densitometric analysis have been performed by Image J Software. The histograms represent the re [file 13046_2022_2459_MOESM10_ESM.jpg]

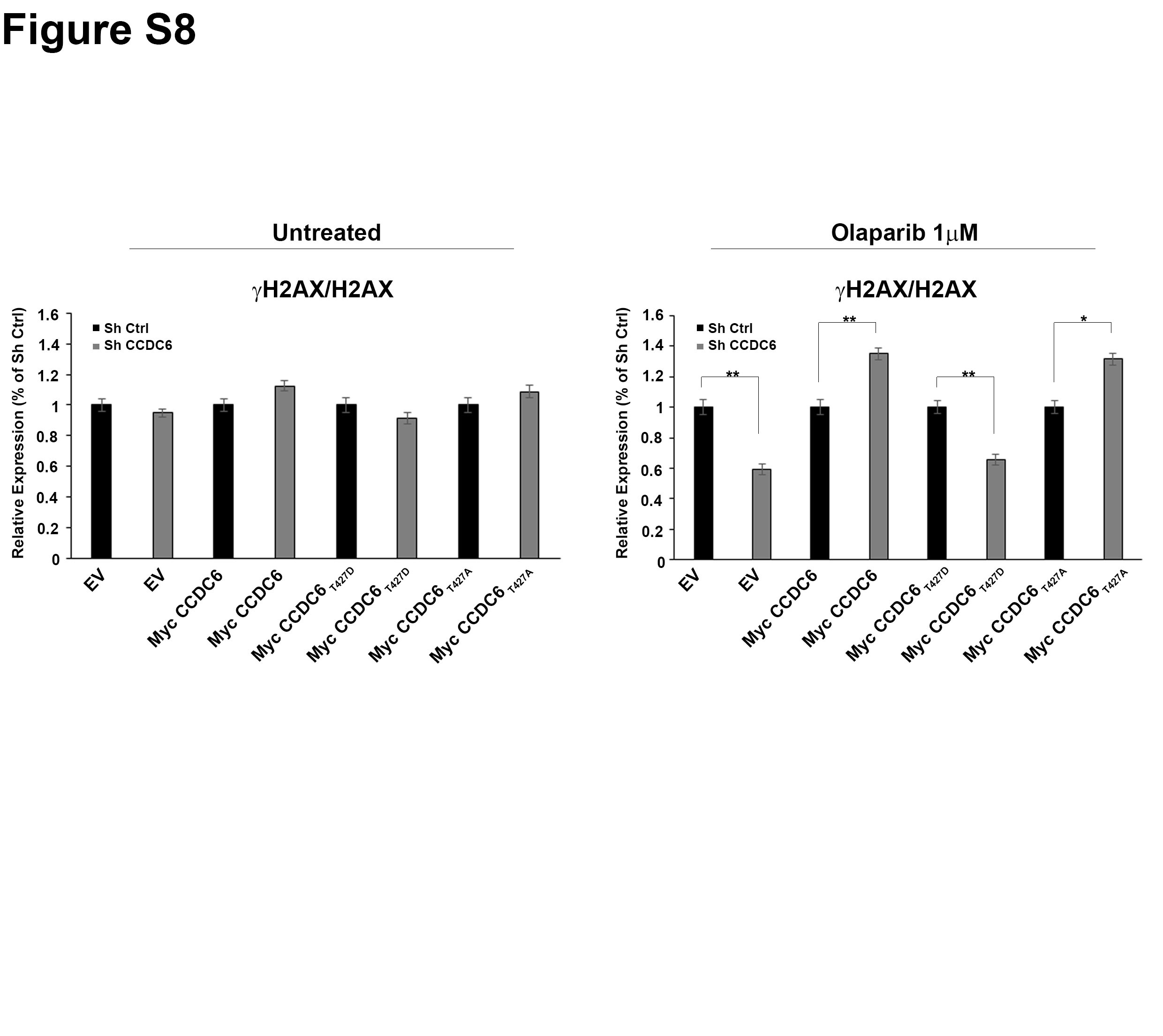

Supplement: Supplementary file 11 — Additional file 11: Figure S8. Densitometric analysis have been performed by Image J Software. The histograms represent the relative protein levels of γH2AX normalised to total H2AX and expressed as relative intensity compared to control. Error bars indicate the measurement of the standard error mean. Statistical significance was verified by 2-tailed Student's t-test (* p <0.05; ** p <0.01 and *** p <0.001). [file 13046_2022_2459_MOESM11_ESM.jpg]

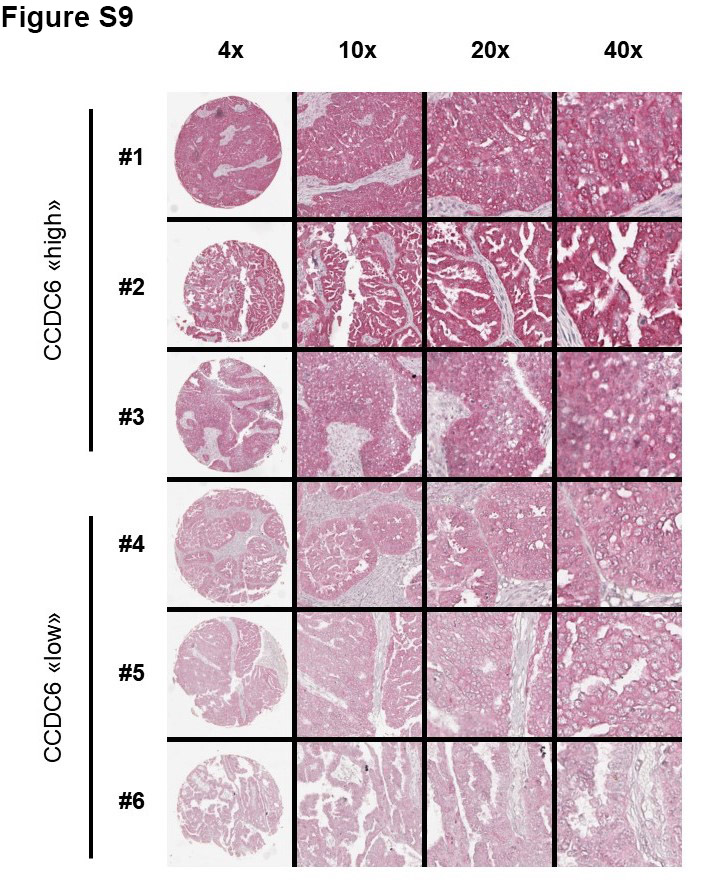

Supplement: Supplementary file 12 — Additional file 12: Figure S9. CCDC6 IHC expression in ovary tumours. Three representative samples of “high” CCDC6 expression (#1, #2, #3) and three representative cases of “low” CCDC6 expression (#4, #5, #6) are shown. For each sample, 10x, 20x, and 40x magnification fields are shown along with the whole TMA core of the digitally scanned glass slide. [file 13046_2022_2459_MOESM12_ESM.jpg]
