# Supplementary material for: The disruption of the CCDC6 – PP4 axis induces a BRCAness like phenotype and sensitivity to PARP inhibitors in high-grade serous ovarian carcinoma
Source: J Exp Clin Cancer Res. 2022 Aug 13;41:245. doi: 10.1186/s13046-022-02459-2 (PMC9375931; doi:10.1186/s13046-022-02459-2)
Supplement: Supplementary file 3 — Additional file 3: Table S2. H-scores of the CCDC6 immunohistochemical staining. [file 13046_2022_2459_MOESM3_ESM.pdf]

Table S2

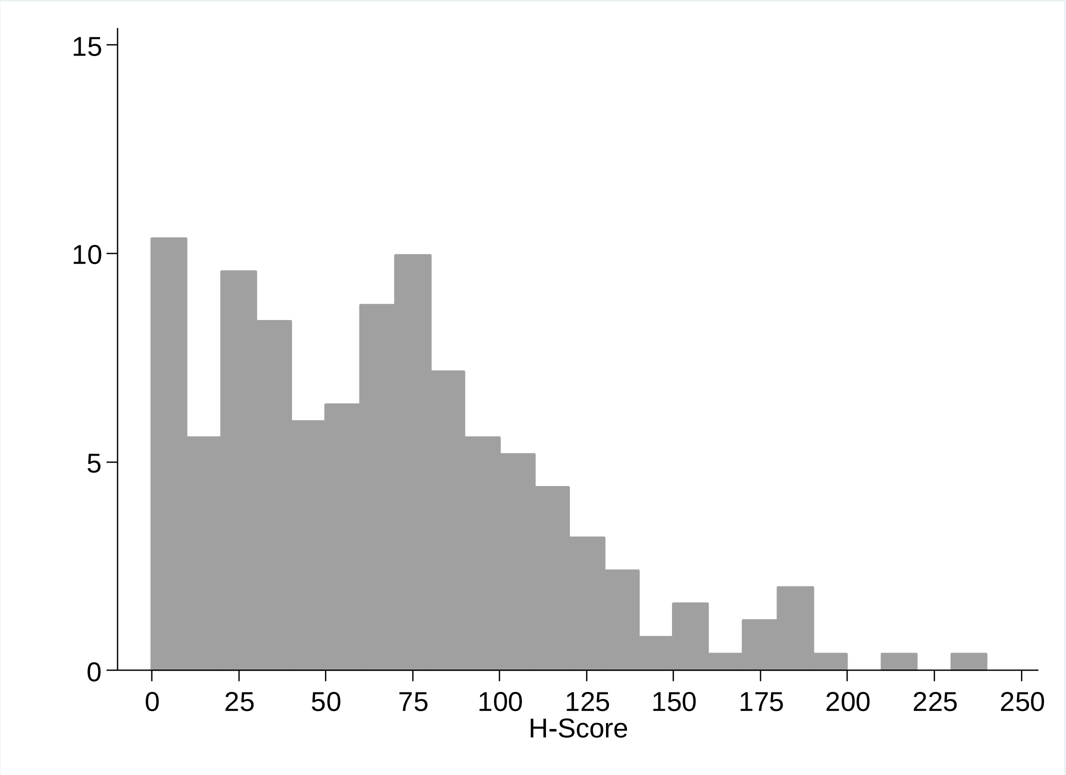

The plot shows the frequency distribution of CCDC6 IHC H-scores as automatically determined by digital image analysis with QuPath software.
