# Supplementary material for: The disruption of the CCDC6 – PP4 axis induces a BRCAness like phenotype and sensitivity to PARP inhibitors in high-grade serous ovarian carcinoma
Source: J Exp Clin Cancer Res. 2022 Aug 13;41:245. doi: 10.1186/s13046-022-02459-2 (PMC9375931; doi:10.1186/s13046-022-02459-2)
Supplement: Supplementary file 4 — Additional file 4: Table S3. Study population of ovarian cancers tested for CCDC6 protein expression. [file 13046_2022_2459_MOESM4_ESM.pdf]

Table S3

	**N=251**
**Age elderly, n(%)**	
<65	220 (87.6%)
≥65	31 (12.4%)
**Tumor histology, n(%)**	
High Grade Serous	159 (63.3%)
Other	81 (32.2%)
unk	11 (4.4%)
**FIGO stage, n(%)**	
I	21 (8.4%)
II	20 (8.0%)
III	171 (68.1%)
IV	39 (15.5%)
**ECOG PS , n(%)**	
0-1	243 (96.8%)
2	8 (3.2%)
**Residual disease, n(%)**	
None	96 (38.2%)
≤ 1 cm	52 (20.7%)
> 1 cm	71 (28.3%)
Not operated	32 (12.7%)
**Treatment, n(%)**	
Carbo-TXL	110 (43.8%)
Carbo-PLD	141 (56.2%)

Summary table of the study population of ovarian cancers tested for CCDC6 protein expression.
